# Atypical functional connectivity in adolescents and adults with persistent and remitted ADHD during a cognitive control task

**DOI:** 10.1038/s41398-019-0469-7

**Published:** 2019-04-12

**Authors:** Giorgia Michelini, Joseph Jurgiel, Ioannis Bakolis, Celeste H. M. Cheung, Philip Asherson, Sandra K. Loo, Jonna Kuntsi, Iman Mohammad-Rezazadeh

**Affiliations:** 1 Social, Genetic and Developmental Psychiatry Centre, Institute of Psychiatry, Psychology and Neuroscience, King’s College London, London, UK; 2 Department of Psychiatry and Behavioral Health, State University New York (SUNY) Stony Brook University, Stony Brook, NY USA; 3 Semel Institute for Neuroscience and Human Behavior, University of California, Los Angeles (UCLA), Los Angeles, CA USA; 4 Department of Biostatistics, Institute of Psychiatry, Psychology and Neuroscience, King’s College London, London, UK; 5 HRL Laboratories, Malibu, CA USA

## Abstract

We previously provided initial evidence for cognitive and event-related potential markers of persistence/remission of attention-deficit/hyperactivity disorder (ADHD) from childhood to adolescence and adulthood. Here, using a novel brain-network connectivity approach, we aimed to examine whether task-based functional connectivity reflects a marker of ADHD remission or an enduring deficit unrelated to ADHD outcome. High-density EEG was recorded in a follow-up of 110 adolescents and young adults with childhood ADHD (87 persisters, 23 remitters) and 169 typically developing individuals during an arrow-flanker task, eliciting cognitive control. Functional connectivity was quantified with network-based graph-theory metrics before incongruent (high-conflict) target onset (pre-stimulus), during target processing (post-stimulus) and in the degree of change between pre-stimulus/post-stimulus. ADHD outcome was examined with parent-reported symptoms and impairment using both a categorical (DSM-IV) and a dimensional approach. Graph-theory measures converged in indicating that, compared to controls, ADHD persisters showed increased connectivity in pre-stimulus theta, alpha, and beta and in post-stimulus beta (all *p* < .01) and reduced pre-stimulus/post-stimulus change in theta connectivity (*p* < .01). In the majority of indices showing ADHD persister–control differences, ADHD remitters differed from controls (all *p* < .05) but not from persisters. Similarly, connectivity measures were unrelated to continuous outcome measures of ADHD symptoms and impairment in participants with childhood ADHD. These findings indicate that adolescents and young adults with persistent and remitted ADHD share atypical over-connectivity profiles and reduced ability to modulate connectivity patterns with task demands, compared to controls. Task-based functional connectivity impairments may represent enduring deficits in individuals with childhood ADHD irrespective of diagnostic status in adolescence/young adulthood.

## Introduction

A coherent communication between brain regions organized in large-scale neural systems, or brain functional connectivity, is thought to have a key role in cognition and behavior^1–4^. Accumulating evidence suggests that atypical connectivity may be implicated in neurodevelopmental disorders^5–7^, such as attention-deficit/hyperactivity disorder (ADHD). In recent years, the study of functional connectivity in ADHD has contributed to the development of neurobiological models highlighting the role of multiple large-scale neural systems in the disorder^8–13^. These include the frontal–parietal network, the ventral–attentional network, and the default-mode network (DMN), involved in executive control, attentional processes, and introspective resting states, respectively^8,9,14^. In particular, it has been hypothesized that ADHD symptomatology may arise from a deviation from neurotypical synchronization and interaction within and between these large-scale networks during brain development^8,12,15,16^. Characterizing the atypical patterns of whole-brain functional connectivity across development may thus provide new insights into ADHD neurobiology.

Most studies to date have investigated functional connectivity in ADHD using resting-state functional magnetic-resonance imaging (fMRI) and reported both reduced^14,16–19^ and increased^5,20–26^ connectivity, for example, within and between the DMN and executive network. Task-based fMRI connectivity studies, which may allow for a more direct characterization of connectivity underlying impaired cognition and behavior^27,28^, have further shown not only hypo-connectivity in fronto-striato-cerebellar networks during sustained attention^29^ and inhibition^30–32^ but also hyper-connectivity within the DMN^30^ and between networks of reward-cognitive control integration^33^ in ADHD. The inconsistencies across previous findings may arise from relatively small samples in most studies and differences in methodology (e.g., region-of-interest vs whole-brain analyses)^12,34^. In addition, fMRI connectivity measures synchronicity between precisely localized networks^5,8,35^ but may not fully capture synchronization between faster brain oscillations underlying fast-changing processes during cognitive tasks.

Investigating functional connectivity using the sub-second temporal resolution of electroencephalography (EEG) instead allows for the measurement of a wider range of brain oscillatory phenomena, including transient changes in connectivity during cognition and behavior^36,37^. Theta, alpha, and beta oscillations during cognitive tasks^4,38–40^, such as flanker tasks^41–45^, have been implicated in processes engaging top–down control networks that require coherent activity between cortically distributed regions. While most EEG connectivity studies on ADHD to date have focused on resting states^46–48^, available task-based connectivity studies in children and adolescents with ADHD indicate not only hyper-connectivity in alpha^49^ and beta^37^ oscillations during attentional tasks but also reduced fronto-parietal theta–alpha connectivity during a flanker task^45,50^. Yet, most EEG connectivity studies on ADHD have used connectivity metrics contaminated by volume-conduction artifacts (i.e., the spreading and mixing of multiple brain sources at the scalp). This methodological limitation can produce inflated connectivity estimates significantly affecting case–control differences^51,52^ and warrants further investigation with metrics uncontaminated by this issue. Furthermore, network approaches based on graph theory have been recently applied to characterize functional connectivity between large-scale brain networks and identify connectivity alterations^2,5,53^. Initial graph-theory evidence from one task-based EEG study showed atypical functional connectivity in children with ADHD^54^, but no study to date has been conducted on adolescents or adults^4,38–45^.

Despite the hypothesis that a deviation from neurotypical large-scale connectivity profiles across development may be implicated in ADHD^8,12,15,16^, little is known on how functional connectivity alterations map onto ADHD developmental outcomes. Longitudinal studies show that ADHD persists, in full or in partial remission, in the majority of adolescents and adults clinically diagnosed in childhood, while a proportion of individuals remit across development^55,56^. Remission of ADHD may be explained in light of compatible neurodevelopmental models^57,58^, which posit that remission may underlie (1) a “normalization” of neural processes (markers of remission) that improve concurrently with clinical symptoms and impairment, whereby individuals with remitted ADHD (ADHD “remitters”) converge toward neurotypical individuals but diverge from individuals with persistent ADHD (ADHD “persisters”)^11,59–61^; and (2) enduring deficits that are unrelated to the clinical outcome, remaining impaired in both ADHD remitters and persisters compared to neurotypical controls^11,62^. The identification of such processes is important for elucidating the neurobiological mechanisms underlying remission/persistence and may point to candidate biomarkers for the development of new interventions. It has been hypothesized that improvements across development in higher-level executive functions would underlie ADHD remission, while persisting impairments in lower-level processes would be displayed regardless of later clinical outcome^11^. Most studies to date, however, found that cognitive performance indices of executive functioning do not distinguish between ADHD persisters and remitters and are thus insensitive to ADHD outcomes^60,61,63–66^. In line with these studies, our recent follow-up study of adolescents and young adults with childhood ADHD show, using a range of attentional, vigilance, and executive paradigms, that cognitive and event-related potential (ERP) markers of executive control (inhibition, working memory, conflict monitoring N2) were insensitive to ADHD outcome^60,61,67^. Instead, cognitive-EEG measures of preparation–vigilance (e.g., reaction-time variability [RTV], target-P3) and error detection (e.g., error-related negativity [ERN] and positivity [Pe]) were markers of remission, distinguishing ADHD remitters from persisters^60,61,67^.

The investigation of task-based functional connectivity across distributed brain networks may provide new insights into the neural pathways of ADHD persistence/remission. The only available task-based connectivity study on persistence/remission reported lower fMRI fronto-thalamic connectivity during response preparation in ADHD persisters compared to remitters and controls during a cued reaction-time task^68^. Despite the complementary benefit of EEG in investigating functional connectivity during cognitive processes^36^, no study to date has examined EEG connectivity in adult ADHD or in relation to remission/persistence.

In the present EEG study, we aimed to investigate brain functional connectivity during a cognitive control task, the arrow flanker task, in adolescents and adults with childhood ADHD and neurotypical controls. We previously reported that, during incongruent (high-conflict) trials of this task, the N2 index of conflict monitoring and cognitive performance measures were insensitive to ADHD outcomes, while error-related ERPs were markers of remission^61^. In this new in-depth analysis of the data presented in our previous study^61^, we sought to take a whole-brain approach to test whether functional connectivity before and during the processing of incongruent stimuli, measured with graph-theory and connectivity metrics not contaminated by volume conduction, is atypical in persistent ADHD, and whether it represents a marker of ADHD remission or an enduring deficit. We hypothesized that both ADHD persisters and remitters would display functional connectivity alterations compared to neurotypical individuals during this task evoking high levels of cognitive control, consistent with most studies examining cognitive and EEG markers of executive processes and ADHD remission^60,61,63–65^.

## Methods

### Sample

The sample consisted of 279 participants who were followed up on average 5.8 years (SD = 1.1) after assessments in childhood^69^, including 110 adolescents and young adults who met Diagnostic and Statistical Manual of Mental Disorders, Fourth Edition (DSM-IV) criteria for combined-type ADHD in childhood (10 sibling pairs and 90 singletons) and 169 control participants (76 sibling pairs and 17 singletons)^60,70^. Participants with ADHD were initially recruited from specialized ADHD clinics and controls from schools in the UK^69^. Exclusion criteria at both assessments were: intelligence quotient (IQ) < 70, autism, epilepsy, brain disorders, and any genetic/medical disorder associated with externalizing behaviors that might mimic ADHD. Among those with childhood ADHD, at follow-up 87 (79%) continued to meet clinical (DSM-IV) levels of ADHD symptoms and impairment (ADHD persisters), while 23 (21%) were below the clinical cut-off (ADHD remitters)^71^ (see “ADHD diagnosis” below). Among ADHD remitters, 14 displayed ≥5 symptoms of inattention or hyperactivity–impulsivity but did not show functional impairment. Participants attended a single research session for clinical, IQ and cognitive-EEG assessments. An estimate of IQ was derived with the vocabulary and block design subtests of the Wechsler Abbreviated Scale of Intelligence^72^. ADHD persisters, remitters, and controls did not differ in age, but there were significantly more males in the remitted group than in the other two groups, with no females among ADHD remitters (Table 1)^60,61^. ADHD persisters showed lower IQ compared to remitters and controls^60,71^. Among participants with childhood ADHD, 47% were on drug treatment at follow-up, but the proportion of participants on medication did not differ between ADHD persisters and remitters (*χ*^2^ = 1.95, *p* = .16)^60^. A 48-h ADHD medication-free period was required before assessments. Parents of all participants gave informed consent following procedures approved by the London-Surrey Borders Research Ethics Committee (09/H0806/58).

**Table 1:** Sample demographics divided by group, with tests for differences between ADHD persisters, remitters, and controls

	ADHD-R (*n* = 23)	ADHD-P (*n* = 87)	Ctrl (*n* = 169)	Group comparison			
					Ctrl vs ADHD-P	Ctrl vs ADHD-R	ADHD-P vs ADHD-R
				*p*	*p*	*p*	*p*
Gender, M:F	23:0	72:15	129:40	.02*	.24	<.01**	.03*
Age, mean (SD)	18.89 (3.06)	18.27 (3.03)	18.77 (2.19)	.15	—	—	—
IQ, mean (SD)	104.57 (13.63)	96.20 (15.33)	109.98 (12.42)	<.01**	<.01**	.10	.02*

Notes: Group differences on gender were tested via Chi-square test; group differences on age and IQ were tested with linear regressions. Group differences in gender, age, and IQ were previously reported in other papers on this sample^60,61^

*ADHD* attention-deficit hyperactivity disorder, *ADHD-P* ADHD persisters, *ADHD-R* ADHD remitters, *Ctrl* Control group, *F* number of females, *M* number of males

**p < .01; *p < .05

### ADHD diagnosis

The Diagnostic Interview for ADHD in Adults (DIVA)^73^ was conducted by trained researchers with parents of the ADHD probands to assess DSM-IV-defined ADHD presence and persistence of the 18 ADHD symptoms. Evidence of impairment commonly associated with ADHD was assessed with the Barkley’s functional impairment scale (BFIS)^74^. Parent-reported DIVA and impairments were used to determine ADHD status, as these were validated against objective markers (cognitive performance and EEG measures) in this sample, whereas the same objective markers showed limited agreement with self-reported ADHD^75^. Childhood ADHD participants were classified as “affected” at follow-up (i.e., ADHD persisters) if they showed ≥6 items in either the inattention or hyperactivity–impulsivity domains on the DIVA and ≥2 areas of impairments on the BFIS; they were classified as remitters otherwise. ADHD outcome was measured using a categorical definition of persistence based on diagnosis (i.e., meeting DSM-IV ADHD diagnostic criteria at follow-up), as well as a dimensional approach based on continuous levels of symptoms of ADHD and impairments, to assess ADHD severity.

### Task

The task was an adaptation of the Eriksen Flanker paradigm designed to increase cognitive load^76^. In each trial, a central fixation mark was replaced by a target arrow (a black 18 mm equilateral triangle). Participants had to indicate whether this arrow pointed toward the left or right by pressing corresponding response buttons with their left or right index fingers. Two flanker arrows identical in shape and size to the target appeared 22 mm above and below the center of the target arrow 100 ms before each target. Both flankers either pointed in the same (congruent) or opposite (incongruent) direction to the target. Cognitive control and conflict monitoring are maximal during incongruent trials. When the target appeared, both target and flankers remained on the screen for 150 ms, with a new trial every 1650 ms. Two-hundred congruent and 200 incongruent trials were arranged in 10 blocks of 40 trials. Only incongruent trials were considered in the present in-depth analysis of the data included in our previous study^61^, as this high-conflict condition has proven more sensitive to ADHD–control differences in previous ERP analyses in this^61^ and other^76,77^ ADHD samples. For further details, see Supplementary Material.

### EEG recording and processing

The EEG was recorded from a 62-channel extended 10–20 system (Brain Products, GmbH, Munich, Germany), using a 500-Hz sampling rate, impedances under 10 kΩ, and recording reference at FCz. The electro-oculograms were recorded from electrodes above and below the left eye and at the outer canthi. Raw EEG recordings were down-sampled to 256 Hz, re-referenced to the average of all electrodes (turning FCz into an active channel), and filtered using Butterworth band-pass filters (0.10–30 Hz, 24 dB/oct). All trials were visually inspected and sections containing electrical or movement artifacts were removed manually. Ocular artifacts were identified using Independent Component Analysis^78^. Sections of data containing artifacts >±100 μV or with a voltage step ≥50 μV were automatically rejected. The artifact-free data were segmented in epochs between −650 and 1000 ms stimulus-locked to incongruent stimuli. Both trials with correct and incorrect responses were examined^61^. Only data containing ≥20 clean segments for condition were included in analyses, leaving 271 participants (83 ADHD persisters, 22 remitters, 166 controls) for correctly-responded trials and 240 (75 ADHD persisters, 20 remitters, 145 controls) for incorrectly-responded trials.

### Connectivity analysis

#### Calculation of functional connectivity

We calculated functional brain connectivity using the imaginary part of coherence (iCoh)^51,79,80^. This measure was chosen to ignore spurious connections between brain signals caused by volume conduction, which can substantially limit the ability to measure functional associations using EEG channels. iCoh captures the non-instantaneous connectivity between brain activities from EEG channels that are phase-lagged (i.e., delay-based)^81,82^. Since volume conduction affects multiple scalp channels with near-zero phase delays, connectivity measured with iCoh is not contaminated by near-instantaneous artifacts of volume conduction. iCoh was measured by isolating the imaginary part of the complex number phase coherence between two signals of same frequency^51^, estimated by calculating their cross-spectrum for each time point with Fast Fourier Transforms using the EEGLAB “newcrossf” function^83^ in Matlab (The Math Works Inc., Natick, MA, USA). iCoh is measured on a scale between 0 and 1. When two signals at the same frequency have identical phase values, possibly due to volume conduction artifacts, iCoh = 0. Instead, if two signals are phase lagged, iCoh > 0^51^. Values of iCoh for all possible electrode pairs (62 × 62) were computed in the theta (4–8 Hz), alpha (8–12 Hz), and beta (12–20 Hz) bands (Supplementary Fig. 1), which have previously been implicated in cognitive processes engaging top–down control networks requiring coherent activity between brain areas^4,38,39^, such as the fronto-parietal network^84–87^.

#### Graph-theory metrics

The high multi-dimensionality of the iCoh measures was disentangled with a graph-theory approach, which allows one to derive global network-based measures and describe functional associations in terms of network properties^2,88,89^. Graph theory is based on mathematical algorithms to quantify the relationships (“edges”) between brain signals from EEG channels, representing the “nodes” of a network. Unthresholded weighted iCoh matrices were used, in line with previous studies^7,90–92^, where each edge is equivalent to the measured iCoh of two electrodes to preserve essential information of a network structure^2,93,94^. Graph-theory metrics measure the degree of network segregation (i.e., the tendency of brain regions to form local clusters with dense functional interconnections) and network integration and efficiency (i.e., the capacity of the network to become interconnected and efficiently exchange information between brain regions)^2,95^. The following commonly used graph measures were calculated^7,54,91,93,96^: average clustering coefficient (the probability of neighboring nodes of being inter-connected, forming densely inter-connected clusters); global efficiency (how efficient the network is in transferring information); and characteristic path length and diameter (respectively, the average number of edges along the shortest paths and the largest possible distance, between all possible pairs of nodes). Graph-theory metrics were computed with the Brain Connectivity^47^ and BioNeCT (https://sites.google.com/site/bionectweb/home; ref. ^3^) toolboxes. In order to examine the modulation of functional connectivity profiles with different conditions and correct vs incorrect performance in this task, we computed connectivity metrics before target (pre-stimulus; −500 to 0 ms) and during target processing (post-stimulus; 0 to 500 ms), as well as separately for correctly- and incorrectly-responded trials.

### Statistical analyses

#### Categorical analysis based on diagnostic status

Connectivity metrics were examined with random-intercept linear models (i.e., multilevel regression models) in Stata 14 (StataCorp, College Station, TX, USA), testing for effects of group (ADHD persisters vs remitters vs controls), time window (pre-stimulus vs post-stimulus), response (correct vs incorrect), and their interaction (group-by-window-by-response). When the three-way interaction was not statistically significant, only statistically significant main effects and two-way interactions were included. For all measures, the within-group degree of change from pre-stimulus to post-stimulus was compared across groups using difference scores to examine how functional connectivity changes with task demands. All models controlled for age and took into account the degree of clustering due to family status. Cohen’s *d* effect sizes are presented along with test statistics, where *d* ≥ 0.20 is a small effect, *d* ≥ 0.50 a medium effect, and *d* ≥ 0.80 a large effect^97^. Given the large number of hypotheses tested, sensitivity analyses applied multiple-testing corrections with false discovery rate (FDR) on post hoc tests with the “multproc” package, using the Simes method, which identifies those tests that remain significant^98^.

Since 80% of our sample consisted of males but groups were not fully matched on sex (Table 1), analyses were performed on the whole sample and then repeated with females (15 ADHD persisters, 41 controls) removed. As in this sample ADHD persisters had a lower IQ than remitters^60^ and childhood IQ predicted ADHD outcome at follow-up^71^, all analyses were also re-run controlling for IQ to examine whether IQ contributes to the results. Finally, even though EEG functional connectivity does not provide a precise localization of functional networks, we examined brain connectivity within and between groups of electrodes from different cortical regions, following previous connectivity studies^99,100^: analyses were repeated using iCoh values within and between clusters of electrodes in different scalp regions (anterior/central/posterior) and between the two hemispheres (left/right) (Supplementary Fig. 3) (for further details, see Supplementary Material).

#### Dimensional analysis with ADHD symptoms/impairment

The association between connectivity metrics and the continua of ADHD symptoms and impairment within individuals with childhood ADHD was examined with random-intercept linear models using DIVA ADHD symptom and impairment scores as independent variables, controlling for age and sex and clustering for family status. Analyses were carried out using standardized scores, thus the beta coefficients are standardized effect sizes comparable to Cohen’s *d*. All analyses were re-run, first, correcting for multiple testing, and, second, controlling for IQ.

#### Association between functional connectivity and cognitive performance

In an additional analysis, we examined the behavioral significance of the EEG connectivity results in relation to task performance. We tested whether functional connectivity measured by mean iCoh was associated with cognitive performance during the incongruent (high-conflict) condition (the same task condition in which connectivity was measured). We previously reported significantly increased mean reaction time (MRT), RTV, and number of errors in ADHD persisters compared to controls in the incongruent condition of this task and intermediate scores with non-significant differences in remitters^61^. The current analyses were restricted to mean iCoh in the pre-stimulus window of correct trials, where differences between ADHD groups and controls were maximal based on categorical analyses. Random-intercept linear models on standardized scores tested the association of mean iCoh in theta, alpha, and beta bands as independent variables with MRT, RTV, and the number of errors as dependent variables. These models were run separately in individuals with childhood ADHD and controls, controlling for age and sex and clustering for family status.

## Results

### Differences between ADHD persisters, remitters, and controls

**Fig. 1:**
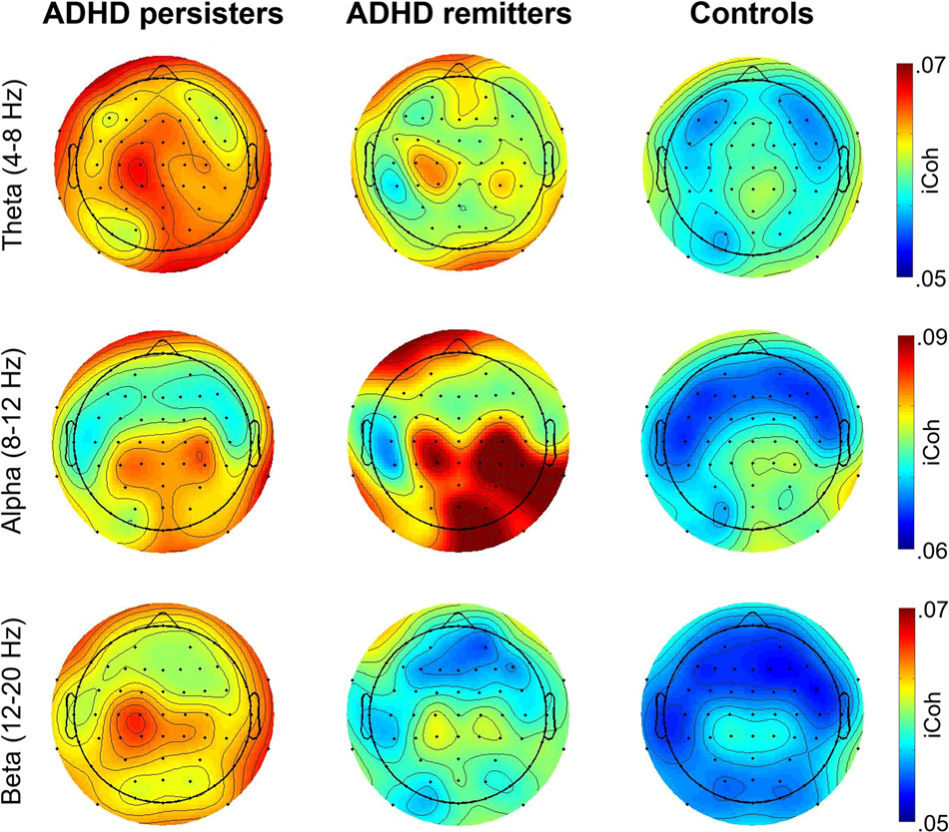
Topographic maps showing the scalp distribution of the imaginary part of coherence (iCoh) in pre-stimulus theta, alpha, and beta frequencies for correctly-responded trials, divided by group (ADHD persisters, remitters, and controls). We calculated the average between iCoh values for each electrode with all other electrodes, which resulted in one average iCoh value for each channel in each participant. By averaging these values across participants within each group, we obtained distribution maps of average connectivity strength between each scalp site and the rest of the scalp for the three groups. The color scale thus represents the average connectivity of each electrode with all other electrodes (higher in red regions, lower in blue regions)

In trials where participants responded correctly, ADHD persisters showed greater clustering coefficient, global efficiency, and mean iCoh and lower path length and diameter compared to controls at all frequency bands in the pre-stimulus window (before target onset) and only in beta in the post-stimulus windows (Table 2, Fig. 1, Supplementary Fig. 2). Similarly, ADHD remitters showed lower pre-stimulus diameter in theta and beta, lower pre-stimulus path length in alpha and beta, and lower post-stimulus diameter in beta, compared to controls. ADHD remitters did not differ from persisters in any connectivity measure in correctly-responded trials, except diameter in beta (where remitters were intermediate between controls and persisters and significantly differed from both groups; Table 2). These findings indicate increased connectivity in both ADHD persisters and remitters compared to controls during correct trials. In trials where an error was made, group differences only emerged for clustering coefficient, global efficiency, and mean iCoh in post-stimulus theta: both ADHD persisters and remitters showed reduced values in these measures (indicating lower connectivity) compared to controls but did not differ from each other (Table 2). All three groups showed increased connectivity (greater clustering coefficient, global efficiency, and mean iCoh; decreased path length and diameter) in trials where an incorrect response occurred, compared to trials with correct responses, in both pre-stimulus and post-stimulus windows (Supplementary Tables 1 and 2), indicating hyper-connectivity before and during incorrect responses. All main and interaction effects are shown in Supplementary Table 2.

**Table 2:** Group comparisons on graph-theory and imaginary coherence measures

		Group comparison						
		Overall group	Ctrl vs ADHD-P		Ctrl vs ADHD-R		ADHD-R vs ADHD-P	
		*p*	*p*	*d*	*p*	*d*	*p*	*d*
Theta								
Average clustering coefficient	Pre, Corr	0.016*	0.004**	*0.63*	0.880	0.29	0.139	0.35
	Pre, Err	0.544	—	—	—	—	—	—
	Post, Corr	0.401	—	—	—	—	—	—
	Post, Err	<0.001***	<0.001***	0.35	0.017*	0.30	0.955	0.05
Global efficiency	Pre, Corr	0.053	0.019*	*0.51*	0.901	0.16	0.145	0.37
	Pre, Err	0.568	—	—	—	—	—	—
	Post, Corr	0.189	—	—	—	—	—	—
	Post, Err	<0.001***	<0.001***	0.35	0.019*	0.30	0.916	0.05
Path length	Pre, Corr	0.012*	<0.001***	*0.58*	0.095	0.30	0.130	0.30
	Pre, Err	0.434	—	—	—	—	—	—
	Post, Corr	0.338	—	—	—	—	—	—
	Post, Err	0.122	—	—	—	—	—	—
Diameter	Pre, Corr	<0.001***	<0.001***	*0.64*	0.012*	0.49	0.352	0.17
	Pre, Err	0.646	—	—	—	—	—	—
	Post, Corr	0.976	—	—	—	—	—	—
	Post, Err	0.279	—	—	—	—	—	—
Mean imaginary coherence	Pre, Corr	0.024*	0.007**	*0.60*	0.952	−0.25	0.140	0.35
	Pre, Err	0.562	—	—	—	—	—	—
	Post, Corr	0.319	—	—	—	—	—	—
	Post, Err	<0.001***	<0.001***	0.35	0.019*	0.30	0.955	0.06
Alpha								
Average clustering coefficient	Pre, Corr	0.001**	<0.001***	0.44	0.097	0.42	0.636	0.06
	Pre, Err	0.415	—	—	—	—	—	—
	Post, Corr	0.328	—	—	—	—	—	—
	Post, Err	0.084	—	—	—	—	—	—
Global efficiency	Pre, Corr	0.003**	0.002**	0.32	0.054	0.39	0.976	0.04
	Pre, Err	0.325	—	—	—	—	—	—
	Post, Corr	0.816	—	—	—	—	—	—
	Post, Err	0.152	—	—	—	—	—	—
Path length	Pre, Corr	<0.001***	<0.001***	0.32	0.005**	0.47	0.539	0.13
	Pre, Err	0.709	—	—	—	—	—	—
	Post, Corr	0.201	—	—	—	—	—	—
	Post, Err	0.235	—	—	—	—	—	—
Diameter	Corr	<0.001***	<0.001***	0.41	0.054	0.30	0.610	0.13
	Err	0.444	—	—	—	—	—	—
Mean imaginary coherence	Pre, Corr	0.001**	<0.001***	0.40	0.073	0.39	0.684	0.04
	Pre, Err	0.341	—	—	—	—	—	—
	Post, Corr	0.501	—	—	—	—	—	—
	Post, Err	0.064	—	—	—	—	—	—
Beta								
Average clustering coefficient	Corr	<0.001***	<0.001***	*0.79*	0.097	*0.51*	0.101	0.31
	Err	0.135	—	—	—	—	—	—
Global efficiency	Corr	<0.001***	<0.001***	*0.73*	0.137	0.44	0.098	0.31
	Err	0.154	—	—	—	—	—	—
Path length	Corr	<0.001***	<0.001***	*0.76*	0.004**	*0.52*	0.090	0.27
	Err	0.343	—	—	—	—	—	—
Diameter	Corr	<0.001***	<0.001***	**0.83**	0.003**	*0.53*	0.044*	0.31
	Err	0.221	—	—	—	—	—	—
Mean imaginary coherence	Corr	<0.001***	<0.001***	*0.77*	0.097	*0.49*	0.101	0.31
	Err	0.135	—	—	—	—	—	—

Notes: Random-intercept linear models tested for main effects of group (ADHD remitters vs ADHD persisters vs controls), time window (pre-stimulus vs post-stimulus) and response (correctly- vs incorrectly-responded trials), two-way interactions (group-by-window, group-by-response, time window-by-response), and three-way interactions (group-by-window-by-response) on connectivity measures. Full results are presented in Supplementary Table 2. Since neither diameter in the alpha band nor any measures in the beta band showed a significant group-by-window interaction, post hoc effects of group were tested for with correctly- and incorrectly-responded trials collapsed across pre-stimulus and post-stimulus time windows. Post hoc comparisons between groups were run only on measures showing a significant overall group effect. Age was also included as a covariate of no interest in all analyses. Data in correctly-responded trials were available for 83 ADHD persisters, 22 remitters, and 166 controls and in incorrectly-responded trials for 75 ADHD persisters, 20 remitters, and 145 controls. *d* ≥ 0.20 = small effect size, *d* ≥ 0.50 = medium effect (in italics), and *d* ≥ 0.80 = large effect size (in bold)

*ADHD* attention-deficit hyperactivity disorder, *ADHD-P* ADHD persisters, *ADHD-R* ADHD remitters, *Corr* trials with correct responses, *Ctrl* Control group, *d* Cohen’s *d* effect size, *Err* trials with incorrect responses, *p* random-intercept linear model significance testing, *Pre* pre-stimulus time window, *Post* post-stimulus time window

*p < 0.05; **p < 0.01; ***p < 0.001

Among measures showing significant group-by-window interactions (all in theta, all except diameter in alpha, none in beta; Supplementary Table 2), significant within-group differences, indicating a change in functional connectivity between the pre-stimulus and the post-stimulus windows, emerged in all groups for all theta connectivity measures; in controls only for clustering coefficient, path length, and mean iCoh in the alpha band; and in both ADHD groups for global efficiency in alpha (Table 3). ADHD persisters and remitters exhibited a significantly lower degree of change compared to controls in all measures of theta connectivity, but no differences emerged between the two ADHD groups (Table 3).

**Table 3:** Within- and between-group effects on measures of change between pre-stimulus and post-stimulus windows in graph-theory and imaginary coherence measures

		Within-group change			Between-group change					
		Ctrl	ADHD-P	ADHD-R	Ctrl vs ADHD-P		Ctrl vs ADHD-R		ADHD-R vs ADHD-P	
		*p*	*p*	*p*	*p*	*d*	*p*	*d*	*p*	*d*
Theta										
Average clustering coefficient	Corr	<0.001***	<0.001***	<0.001***	0.001**	0.42	0.010*	0.41	0.981	0.05
	Err	<0.001***	<0.001***	<0.001***	0.011*	0.33	0.618	0.06	0.370	0.26
Global efficiency	Corr	<0.001***	<0.001***	<0.001***	0.002**	0.40	0.014*	0.38	0.997	0.04
	Err	<0.001***	<0.001***	<0.001***	0.017*	0.31	0.643	0.06	0.400	0.25
Path length	Corr	<0.001***	<0.001***	<0.001***	<0.001***	*0.61*	0.014*	0.44	0.506	0.19
	Err	<0.001***	<0.001***	<0.001***	0.058	0.27	0.776	0.11	0.209	0.36
Diameter	Corr	<0.001***	<0.001***	<0.001***	<0.001***	*0.61*	0.016*	0.43	0.499	0.19
	Err	<0.001***	<0.001***	<0.001***	0.058	0.20	0.776	0.14	0.209	0.33
Mean imaginary coherence	Corr	<0.001***	<0.001***	<0.001***	0.001**	0.42	0.011*	0.40	0.995	0.05
	Err	<0.001***	<0.001***	<0.001***	0.013*	0.33	0.632	0.06	0.378	0.26
Alpha										
Average clustering coefficient	Corr	0.002**	0.910	0.767	0.055	0.27	0.091	0.38	0.704	0.09
	Err	0.001**	0.981	0.599	0.069	0.28	0.267	0.16	0.468	0.13
Global efficiency	Corr	0.728	0.004**	0.045*	0.071	0.27	0.147	0.40	0.705	0.11
	Err	0.155	0.029*	0.683	0.019*	0.38	0.140	0.25	0.389	0.15
Path length	Corr	0.002**	0.856	0.319	0.124	0.20	0.049*	0.42	0.349	0.23
	Err	0.011*	0.831	0.931	0.023*	0.37	0.094	0.33	0.743	0.07
Mean imaginary coherence	Corr	0.020*	0.491	0.472	0.064	0.27	0.111	0.37	0.735	0.08
	Err	0.001**	0.545	0.791	0.015*	0.40	0.087	0.30	0.469	0.13

Notes: Random-intercept linear models tested for main effects of group (ADHD remitters vs ADHD persisters vs controls), time window (pre-stimulus vs post-stimulus) and response (correctly- vs incorrectly-responded trials), two-way interactions (group-by-window, group-by-response, time window-by-response), and three-way interactions (group-by-window-by-response) on connectivity measures. Full results are presented in Supplementary Table 2. Post hoc tests on within- and between-group effects of change were run only on measures showing a significant group-by-window interaction. Since this interaction was not significant in diameter in the alpha band or in any measures in the beta band, post-hoc within- and between-groups effects of change were not tested. Age was also included as a covariate of no interest in all analyses. Data in correctly-responded trials were available for 83 ADHD persisters, 22 remitters, and 166 controls; and in incorrectly-responded trials for 75 ADHD persisters, 20 remitters, and 145 controls. *d* ≥ 0.20 = small effect size, *d* ≥ 0.50 = medium effect (in italics), *d* ≥ 0.80 = large effect

*ADHD* attention-deficit hyperactivity disorder, *ADHD-P* ADHD persisters, *ADHD-R* ADHD remitters, *Corr* trials with correct responses, *Ctrl* Control group, *d* Cohen’s *d* effect size, *Err* trials with incorrect responses, *p* random-intercept linear model significance testing

*p < 0.05; **p < 0.01; ***p < 0.001

Multiple-testing corrections (controlling the FDR at 15%) on post hoc group comparisons (separately for ADHD persisters vs controls, ADHD remitters vs controls, ADHD persisters vs remitters) showed that all statistically significant differences between controls and ADHD remitters, and between controls and ADHD persisters remained significant. The only significant difference between ADHD persisters and remitters (in beta diameter) was no longer significant when correcting for multiple testing. All significant group differences on measures of pre-stimulus/post-stimulus change remained significant after multiple-testing corrections.

All results remained unchanged when rerunning analyses on the male-only sample (Supplementary Tables 3 and 4), except that the *p* values of certain tests that were statistically significant in the full sample became trend-level effects (*p* = 0.05–0.10). All effect sizes were similar to those on the full sample, suggesting that these non-significant results may be due to lower power in this smaller sample.

Results of group comparisons on connectivity measures in pre- and post-stimulus were largely unchanged when IQ was included as a covariate in categorical analyses (Supplementary Table 5). A few differences between persisters and controls on measures of pre-stimulus/post-stimulus change in theta and alpha connectivity during error trials were no longer significant (Supplementary Table 6).

Results of analyses on group differences in local connectivity within and between cortical regions were consistent with those on whole-brain connectivity, indicating that functional connectivity profiles were not driven by stronger connectivity within or between particular regions. Specifically, group comparisons showed the same pattern of results when considering functional connectivity within more localized cortical regions (within anterior, central, and posterior regions and within left and right hemispheres), between antero-central, centro-posterior, and antero-posterior regions, and between the two hemispheres (for full results, see Supplementary Material).

### Association with ADHD symptoms and impairment

In dimensional analyses on participants with childhood ADHD, no association emerged between ADHD symptoms and any connectivity measure in theta, alpha, or beta frequencies in correct or error trials (Table 4). Functional impairment was not associated with any connectivity measure in the theta band but showed associations with a subgroup of measures in alpha and beta in correct and error trials (Table 4). Results remained largely unchanged when controlling for IQ (Supplementary Table 7). Statistically significant associations that emerged with ADHD impairment were no longer significant after applying multiple-testing corrections.

**Table 4:** Dimensional associations of graph-theory and imaginary coherence measures with interview-based DIVA ADHD symptom counts and clinical impairment within the ADHD group only (*n* = 110), controlling for age and gender

		ADHD symptoms		Impairment	
		*β* (95% CIs)	*p*	*β* (95% CIs)	*p*
Theta					
Average clustering coefficient	Pre, Corr	−0.005 (−0.202; 0.193)	0.964	0.160 (−0.065; 0.384)	0.163
	Pre, Err	0.020 (−0.177; 0.216)	0.844	0.178 (−0.040; 0.398)	0.110
	Post, Corr	−0.021 (−0.218; 0.174)	0.827	0.176 (−0.0041; 0.393)	0.111
	Post, Err	−0.088 (−0.259; 0.084)	0.315	−0.068 (−0.263; 0.127)	0.494
Global efficiency	Pre, Corr	−0.037 (−0.216; 0.142)	0.685	0.089 (−0.115; 0.292)	0.393
	Pre, Err	0.004 (−0.181; 0.189)	0.969	0.167 (−0.280; 0.373)	0.110
	Post, Corr	−0.043 (−0.267; 0.152)	0.667	0.145 (−0.074; 0.365)	0.194
	Post, Err	−0.111 (−0.279; 0.057)	0.196	−0.152 (−0.344; 0.040)	0.120
Path length	Pre, Corr	0.033 (−0.145; 0.211)	0.716	−0.123 (−0.322; 0.076)	0.226
	Pre, Err	−0.027 (−0.231; 0.178)	0.797	−0.175 (−0.402; 0.053)	0.132
	Post, Corr	0.067 (−0.140; 0.273)	0.528	−0.100 (−0.332; 0.131)	0.395
	Post, Err	0.108 (−0.092; 0.308)	0.290	0.056 (−0.171; 0.282)	0.630
Diameter	Pre, Corr	0.049 (−0.135; 0.232)	0.601	−0.109 (−0.321; 0.102)	0.310
	Pre, Err	−0.043 (−0.251; 0.165)	0.685	−0.168 (−0.107; 0.028)	0.153
	Post, Corr	0.030 (−0.161; 0.222)	0.756	−0.116 (−0.329; 0.098)	0.287
	Post, Err	0.100 (−0.100; 0.300)	0.328	0.020 (−0.202; 0.242)	0.861
Mean imaginary coherence	Pre, Corr	0.013 (−0.205; 0.180)	0.898	0.142 (−0.077; 0.361)	0.204
	Pre, Err	0.015 (−0.178; 0.208)	0.878	0.175 (−0.040; 0.390)	0.110
	Post, Corr	−0.028 (−0.224; 0.168)	0.778	0.167 (−0.051; 0.385)	0.134
	Post, Err	−0.096 (−0.266; 0.074)	0.268	−0.101 (−0.295; 0.093)	0.306
Alpha					
Average clustering coefficient	Pre, Corr	0.014 (−0.186; 0.215)	0.894	0.044 (−0.186; 0.274)	0.708
	Pre, Err	0.051 (−0.130; 0.233)	0.578	0.157 (−0.049; 0.363)	0.135
	Post, Corr	0.064 (−0.121; 0.249)	0.500	0.256 (0.056; 0.456)	0.012*
	Post, Err	0.117 (−0.063; 0.297)	0.204	0.223 (0.017; 0.429)	0.034*
Global efficiency	Pre, Corr	−0.027 (−0.231; 0.176)	0.794	0.091 (−0.326; 0.145)	0.450
	Pre, Err	0.033 (−0.160; 0.226)	0.738	0.130 (−0.089; 0.349)	0.245
	Post, Corr	0.052 (−0.125; 0.230)	0.563	0.199 (0.004; 0.394)	0.046*
	Post, Err	0.108 (−0.063; 0.280)	0.216	0.222 (0.021; 0.419)	0.031*
Path length	Pre, Corr	<0.001 (−0.184; 0.183)	0.998	0.080 (−0.129; 0.289)	0.452
	Pre, Err	−0.026 (−0.219; 0.167)	0.793	−0.132 (−0.347; 0.083)	0.229
	Post, Corr	−0.052 (−0.237; 0.132)	0.580	−0.202 (−0.404; 0.000)	0.050
	Post, Err	−0.131 (−0.319; 0.057)	0.172	−0.222 (−0.436; −0.008)	0.042*
Diameter	Pre, Corr	−0.027 (−0.223; 0.129)	0.784	−0.051 (−0.277; 0.175)	0.659
	Pre, Err	−0.080 (−0.272; 0.113)	0.417	−0.177 (−0.397; 0.043)	0.114
	Post, Corr	−0.053 (−0.245; 0.139)	0.588	−0.232 (−0.442; −0.023)	0.030*
	Post, Err	−0.134 (−0.332; 0.064)	0.185	−0.201 (−0.428; 0.027)	0.083
Mean imaginary coherence	Pre, Corr	0.003 (−0.202; 0.195)	0.973	−0.003 (−0.229; 0.224)	0.981
	Pre, Err	0.049 (−0.152; 0.251)	0.631	0.165 (−0.063; 0.393)	0.156
	Post, Corr	0.062 (−0.121; 0.245)	0.505	0.242 (0.045; 0.441)	0.016*
	Post, Err	0.115 (−0.063; 0.294)	0.204	0.223 (0.018; 0.427)	0.033*
Beta					
Average clustering coefficient	Pre, Corr	0.124 (−0.100; 0.349)	0.278	0.306 (0.062; 0.550)	0.014*
	Pre, Err	0.051 (−0.149; 0.252)	0.613	0.203 (−0.022; 0.429)	0.077
	Post, Corr	0.093 (−0.141; 0.328)	0.435	0.264 (0.001; 0.527)	0.049*
	Post, Err	0.045 (−0.160; 0.250)	0.666	0.166 (−0.062; 0.393)	0.153
Global efficiency	Pre, Corr	0.100 (−0.119; 0.319)	0.372	0.299 (0.061; 0.536)	0.014*
	Pre, Err	0.047 (−0.155; 0.248)	0.650	0.210 (−0.016; 0.436)	0.069
	Post, Corr	0.068 (−0.167; 0.302)	0.572	0.248 (−0.015; 0.512)	0.065
	Post, Err	0.042 (−0.163; 0.248)	0.688	0.166 (−0.062; 0.394)	0.152
Path length	Pre, Corr	−0.092 (−0.289; 0.105)	0.361	−0.233 (−0.450; −0.016)	0.035*
	Pre, Err	−0.067 (−0.264; 0.131)	0.508	−0.198 (−0.419; 0.023)	0.080
	Post, Corr	−0.073 (−0.280; 0.134)	0.490	−0.191 (−0.425; 0.043)	0.110
	Post, Err	−0.080 (−0.283; 0.124)	0.444	−0.163 (−0.387; 0.061)	0.153
Diameter	Pre, Corr	−0.118 (−0.318; 0.083)	0.251	−0.253 (−0.474; −0.033)	0.024*
	Pre, Err	−0.094 (−0.301; 0.112)	0.372	−0.166 (−0.395; 0.063)	0.157
	Post, Corr	−0.105 (−0.312; 0.102)	0.320	−0.190 (−0.425; 0.045)	0.114
	Post, Err	−0.089 (−0.299; 0.122)	0.410	−0.124 (−0.357; 0.108)	0.294
Mean imaginary coherence	Pre, Corr	0.118 (−0.105; 0.341)	0.301	0.305 (0.063; 0.548)	0.013*
	Pre, Err	0.051 (−0.150; 0.251)	0.620	0.207 (−0.019; 0.432)	0.072
	Post, Corr	0.085 (−0.150; 0.321)	0.478	0.259 (−0.005; 0.523)	0.054
	Post, Err	0.044 (−0.162; 0.249)	0.676	0.166 (−0.062; 0.394)	0.153

Notes: Random-intercept linear models tested for the effect of ADHD symptom count/impairment on each connectivity measure. *β* ≥ 0.20 = small effect size, *β* ≥ 0.50 = medium effect, *β* ≥ 0.80 = large effect. Data in correctly-responded trials were available for 105 childhood ADHD participants (83 ADHD persisters, 22 remitters) and in incorrectly-responded trials for 95 childhood ADHD participants (75 ADHD persisters, 20 remitters)

*ADHD* attention-deficit/hyperactivity disorder, *β* standardized regression coefficient, *CI* confidence interval, *Corr* trials with correct responses, *DIVA* Diagnostic Interview for ADHD in Adults, *Err* trials with incorrect responses, *p* random-intercept linear model significance testing, *Pre* pre-stimulus time window, *Post* post-stimulus time window

*p < 0.05

### Association with cognitive performance

Increased functional connectivity was associated with worse cognitive performance. Specifically, greater mean iCoh in the theta and beta bands showed statistically significant effects on greater RTV in the childhood ADHD group and on greater number of errors in both the childhood ADHD and control groups (Supplementary Table 8). Alpha mean iCoh was not significantly associated with any performance measure in either group. None of the pre-stimulus iCoh connectivity measures had a significant effect on MRT in either group.

## Discussion

Using a network-based EEG functional connectivity approach during an arrow flanker task, our results show widespread hyper-connectivity underlying cognitive control processes, as well as reduced adjustments of connectivity with changed task demands, in individuals with persistent ADHD compared to neurotypical controls. ADHD remitters showed connectivity impairments similar to persisters and differed from controls in most measures of connectivity and of connectivity adjustments. These findings indicate that hyper-connectivity and reduced ability to modulate connectivity patterns with task demands characterize adolescents and young adults with both persistent and remitted ADHD. Atypical functional connectivity during cognitive control processes may thus represent an enduring deficit in adolescents and adults with childhood ADHD, irrespective of their diagnostic outcome.

Two main connectivity impairments emerged in individuals with persistent ADHD compared to controls. First, ADHD persisters showed increased global connectivity (higher iCoh), network clustering (higher clustering coefficient), efficiency (higher global efficiency), and integration (lower path length and diameter) at all frequency bands prior to target onset in trials with correct behavioral responses, as well as during target processing in beta oscillations. This increased task-based functional connectivity is consistent with a previous EEG study reporting pre-target over-connectivity in children with ADHD^37^. More generally, these findings align with previous EEG and fMRI evidence indicating hyper-connectivity in individuals with ADHD during task performance^30,33,49^, but not with other studies, mainly with fMRI, showing task-based hypo-connectivity^30–32^. Some of these inconsistencies may arise from methodological differences between fMRI and EEG, which provide complementary pictures of functional connectivity, especially during cognitive tasks: the former on slower oscillations^35^ and the latter on faster rhythms. Our results suggest that ADHD persisters exhibited hyper-connectivity in theta, alpha, and beta oscillations prior to the onset of incongruent stimuli in this cognitive control task, as well as in beta specifically during target processing. Connectivity in these oscillations during cognitive tasks has been associated with cognitive processes engaging control networks and requiring coordination of activity between distributed and large-scale brain networks^4,38,39^. Here hyper-connectivity in these oscillations in persistent ADHD may reflect exaggerated interactions between brain regions, both during the inactive pre-stimulus period and during cognitive target processing. Considering the high cognitive demands induced by incongruent stimuli in this highly effortful task, which requires a response at every trial, these findings may reflect hyper-connectivity in distributed brain networks underlying higher-level cognitive functions. Second, while all groups showed a significant increase in theta connectivity in changing from pre-stimulus to post-stimulus windows following onset of incongruent stimuli, this change was reduced in ADHD persisters compared to controls. This result in individuals with ADHD may point to a reduced ability to modulate brain connectivity patterns in slow oscillations from a relatively inactive context to a condition requiring cognitive control and conflict monitoring. This finding is in line with previous reports indicating reduced regulation of brain activity in ADHD between different cognitive states^101–103^. Overall, these findings show widespread connectivity impairments underlying cognitive control processes in ADHD persisters and advance our understanding of the neural underpinnings of persistent ADHD in adolescence and early adulthood.

Our study represents the first investigation into EEG connectivity in adolescents and adults with remitted ADHD. In several functional connectivity measures sensitive to impairments in persisters, ADHD remitters were impaired compared to controls and indistinguishable from persisters, consistent with our hypotheses. ADHD remitters also showed the same reduction in all measures of pre-stimulus/post-stimulus change in theta connectivity displayed by persisters. As such, brain connectivity impairments during this cognitive control task were insensitive to ADHD remission/persistence in adolescence and early adulthood and may represent enduring deficits irrespective of current diagnostic status. Findings from dimensional analyses on ADHD severity supported these results, as most connectivity measures in participants with childhood ADHD were unrelated to continuous levels of ADHD symptoms and impairments. Of note, while results of categorical analyses were largely unchanged after correcting for multiple testing, the few significant associations between connectivity and functional impairment (all with small effect sizes) did not survive multiple-testing corrections. These connectivity findings are consistent with previous cognitive-EEG studies, including our previous analyses on this sample^60,61^, reporting that executive-functioning measures are insensitive to ADHD outcomes in adolescence and adulthood^60,61,63–65^. They further extend our earlier findings of no differences between ADHD remitters and persisters on the N2 (reflecting conflict monitoring) and indices of cognitive performance in the same incongruent condition^61^. More broadly, our findings support the co-existence of separate neurobiological processes underpinning developmental pathways to remission and persistence of ADHD^57^: functional connectivity during high-conflict trials in this cognitive control task appears unrelated to clinical outcome, unlike measures of less-effortful, non-executive processes (e.g., preparation–vigilance) identified as markers of remission in our previous cognitive-EEG studies^60,61,67^ and in fMRI studies^68,104^. A clinical implication is that connectivity impairments underlying executive-control processes may not be suitable targets for interventions or objective indicators of treatment monitoring, consistent with previous evidence of no effects of stimulants on EEG connectivity in ADHD^47,105^. Future studies should examine whether EEG functional connectivity during less effortful activities, such as attentional processes, represent markers of remission and candidate targets for new treatments, similar to measures of non-executive processes in our previous studies^60,61,67^.

Of note, while widespread group differences emerged in correctly-responded trials, where participants successfully overcame the conflict generated by incongruent target and flanking stimuli, group differences in error trials emerged only in three measures of post-stimulus theta connectivity. An incorrect response likely represents a failure of cognitive control, required for selection of a correct response in the highly challenging incongruent condition. The limited group differences in connectivity during incorrect responses may suggest that a suboptimal pattern of functional connectivity may attenuate the differences in brain-network profiles between neurotypical individuals and individuals with ADHD, who are prone to making more incorrect responses in this task^61^. In addition, in all groups functional connectivity was increased during incorrect responses compared to correct responses, both during the inactive pre-stimulus window and during processing of incongruent stimuli. This is in line with the interpretation that hyper-connectivity displayed in the ADHD groups was dysfunctional during this task. An additional analysis testing whether hyper-connectivity was related to impairments in cognitive performance during this task further confirmed this pattern, as pre-stimulus hyper-connectivity in theta and beta oscillations was associated with fewer correct responses in individuals with childhood ADHD and controls and with increased RTV in individuals with childhood ADHD. Increased functional connectivity in both ADHD persisters and remitters may thus contribute to the lack of differences in cognitive performance measures between ADHD remitters and persisters reported in our previous study^61^. Overall, a suboptimal pattern of hyper-connectivity underlying cognitive control processes may lead to dysfunctional behavioral responses, both in neurotypical individuals and in individuals with childhood ADHD.

A limitation of this study is that, despite the large sample, the low ADHD remission rate at follow-up resulted in a relatively small group of remitters. Therefore, we could not exclude the possibility that some non-significant group differences could be due to low power. However, the moderate effect sizes (*d* = 0.38–0.53) between ADHD remitters and controls, but negligible or small (*d* = 0.02–0.36) between remitters and persisters, in measures showing ADHD persister–control differences suggest that we had sufficient power to detect, with the current sample sizes, differences in connectivity with at least moderate effect sizes. In addition, our sample included young adults as well as adolescents who are still undergoing rapid cortical maturation. While analyses controlled for age, future follow-up assessments with participants having reached adulthood could provide further insight into developmental patterns. Finally, the relatively poor spatial resolution of scalp-EEG did not allow precise localization of the brain networks. Yet, the current EEG connectivity analyses allowed precise temporal resolution during two short time windows and both correct and incorrect behavioral responses, as well as connectivity estimates unaffected by volume-conduction artifacts and examination of whole-brain network properties. The results of local connectivity within and between cortical regions were further consistent with those of whole-brain analyses, indicating comparable effects in more localized networks.

In conclusion, we report new evidence of shared atypical task-based connectivity profiles in adolescents and young adults with persistent and remitted ADHD. These connectivity alterations may represent enduring deficits and neural signatures associated with having a history of childhood ADHD, but appear unrelated to follow-up diagnostic status. Connectivity impairments underlying executive processes may represent associated characteristics or risk factors in ADHD^10^, which do not follow the developmental pathways of clinical profiles. Future studies should explore the presence of potential compensatory mechanisms that may enable developmental improvements in clinical profiles and non-executive cognitive processes in individuals with remitted ADHD^60,61,67^, despite enduring functional connectivity alterations during cognitive control.

## Supplementary information

Supplementary Material.
