# Supplementary material for: Atypical functional connectivity in adolescents and adults with persistent and remitted ADHD during a cognitive control task
Source: Transl Psychiatry. 2019 Apr 12;9:137. doi: 10.1038/s41398-019-0469-7 (PMC6461684; doi:10.1038/s41398-019-0469-7)
Supplement: Supplementary file 1 — Supplementary Material. [file 41398_2019_469_MOESM1_ESM.docx]

**Supplementary material**

***Atypical functional connectivity in adolescents and adults with persistent and remitted ADHD during a cognitive control task***

*Giorgia Michelini, Joseph Jurgiel, Ioannis Bakolis, Celeste H. M. Cheung, Philip Asherson, Sandra K. Loo, Jonna Kuntsi, Iman Mohammad-Rezazadeh*

**Further details on the task**

The version of Eriksen flanker task used in this study consisted of 10 blocks of 40 trials where congruent versus incongruent conditions and the direction of responses (left versus right) were counter-balanced and randomized. Participants were seated on an adjustable chair in an acoustically shielded, video-monitored room. Two practice blocks were administered before the task blocks and task comprehension was ascertained prior to task performance. On congruent trials, flanker and target arrowheads pointed in the same direction; on incongruent trials, they pointed in opposite directions. Flankers were presented every 1650 ms (inter-trial interval (ITI), therefore an inter-block interval (IBI) was 1400 ms). ITI and IBI were fixed. After each block, feedback was presented on screen to emphasise both speed and accuracy, to encourage participants to make enough errors to enable analysis of trials with incorrect responses, and enough correct responses for analysis of correct responses. Where participants made >10% errors on congruent or >40% errors on incongruent trials, they were instructed to slow down. Where participants made <10% errors on congruent or <40% errors on incongruent trials, they were instructed to perform faster. If neither rule applied, feedback informed participants to continue the same way. The task was run during a 1.5-hour recording session between two other tasks which are not reported here: preceded by a Continuous Performance Test-OX and followed by the Fast task (see^1^ for details). Breaks of at least 5 minutes were given in between tasks. When necessary, participants were told to minimize movement or blinking.

**Results in the male-only sample**

The majority of individuals in our sample (80%) were males. Since groups were not fully matched on sex (Table 1), analyses were repeated with females (15 ADHD persisters, 41 controls) removed. Results were largely unchanged when rerunning analyses on the male-only sample (Supplementary Tables 3-4). Only a few tests that were significant in the full sample on connectivity in the theta band became trend-level effects (p=0.05-0.10) in males only: the comparison between ADHD persisters and controls in pre-stimulus global efficiency in correct responses, and the comparison between ADHD remitters and controls in pre-stimulus diameter in correct responses and in post-stimulus average clustering coefficient, global efficiency and iCoh in error responses (Supplementary Table 3). When repeating the analyses of within- and between-group change between time windows, differences between ADHD persisters and controls on the change between time windows in average clustering coefficient, global efficiency and mean iCoh during error trials, as well as between ADHD remitters and controls in theta diameter in correct trials and in alpha path length in correct trials, were no longer significant (Supplementary Table 4).

**Results covarying for IQ**

ADHD persisters in this sample had a lower IQ than ADHD remitters and controls^1^, and childhood IQ predicted ADHD outcome at follow up^2^. To examine whether IQ contributes to the differences between groups in brain connectivity, all analyses were re-run controlling for IQ. Results of group comparisons in the pre-stimulus and post-stimulus windows for correct and error responses remained the same when controlling for IQ (Supplementary Table 5), with the only exceptions being that the comparisons between ADHD remitters and controls in alpha pre-stimulus global efficiency and alpha diameter in correct trials became significant (while they were at trend-level in the main analyses). When repeating the analyses of within- and between-group change between time windows covarying for IQ, differences between ADHD persisters and controls on the change between time windows in theta average clustering coefficient, global efficiency and mean iCoh during error trials, and in alpha global efficiency, path length and mean iCoh during error trials were no longer significant (Supplementary Table 6).

When controlling for IQ in dimensional analyses, the results on the association between connectivity measures and DIVA ADHD symptoms (revealing no significant associations in the main analyses) remained unchanged. The statistically significant associations of impairment with post-stimulus global efficiency in the alpha band and pre-stimulus path length in the beta band in correct trials were no longer significant (at trend-level) when controlling for IQ, while the non-significant (trend-level) associations of impairment with pre-stimulus average clustering coefficient, global efficiency, path length and mean iCoh in the beta band in incorrect trials became significant (Supplementary Table 7).

**Results of connectivity within and between cortical regions**

In addition to whole-brain connectivity analysis, we also examined patterns of local brain connectivity within cortical regions. Local connectivity was quantified with mean iCoh values between groups of electrodes. First, we computed mean iCoh within anterior (AF3, AF4, Fz, F1, F2, F3, F4, F5, F6, F7, F8, AF7, AF8), central (Cz, C1, C2, C3, C4, C5, C6, FCz, FC1, FC2, FC3, FC4, FC5, FC6, CPz, CP1, CP2, CP3, CP4, CP5, CP6), and posterior (Pz, P3, P4, P7, P8, PO7, PO8, PO3, PO4, POz, Oz, O1, O2) scalp regions. Secondly, we analysed connectivity within left (AF3, AF7, F1, F3, F5, F7, FC1, FC3, FC5, FT7, FT9, C1, C3, C5, T7, CP1, CP3, CP5, TP7, TP9, P3, P7, PO3, PO7, PO9, O1) and right (AF4, AF8, F2, F4, F6, F8, FC2, FC4, FC6, FT8, FT10, C2, C4, C6, T8, CP2, CP4, CP6, TP8, TP10, P4, P8, PO4, PO8, PO10, O2) hemispheres (Supplementary Figure 3). Both groups of analyses focused on correctly-responded trials only, where group differences where maximal. For the analysis of connectivity within each local region, random intercept linear models tested for main effects of group (ADHD persisters vs ADHD remitters vs controls), time window (pre-stimulus vs post-stimulus) and region (anterior vs central vs posterior region; left vs right hemisphere), and three-way group-by-window-by-region interactions on mean iCoh in the theta, alpha and beta bands separately. For the analysis of connectivity between anterior/central/posterior regions, random intercept linear models tested for main effects of group (ADHD persisters vs ADHD remitters vs controls), time window (pre-stimulus vs post-stimulus) and region (anterio-central vs anterio-posterior vs centro-posterior connectivity), and for three-way group-by-window-by-region interactions on mean iCoh between each of these regions in the theta, alpha and beta bands separately. Finally, for the analysis of connectivity between right and left hemispheres, random intercept linear models tested for main effects of group (ADHD persisters vs ADHD remitters vs controls) and time window (pre-stimulus vs post-stimulus), and for two-way group-by-window interactions on mean iCoh measured between the two hemispheres in the theta, alpha and beta bands separately.

For iCoh within anterior/central/posterior cortical regions, the three-way group-by-window-by-region and two-way group-by-region interactions were not significant for theta, alpha or beta (all p>0.23). These results indicate that the pattern of group differences did not vary in the three local regions. For beta, the two-way group-by-window interaction was further not significant (p=0.50), indicating that the group differences did not vary in the two time windows, in line with the whole-brain analysis. After removing these non-significant interactions, results of analyses on group differences in local connectivity within cortical regions were consistent with those on whole-brain connectivity. Specifically, post-hoc group comparisons showed that, for theta and alpha bands in the pre-stimulus window, ADHD persisters had significantly greater values of iCoh than controls (p<0.02) but did not differ from remitters (p>0.21). Relative to controls, remitters showed no differences in pre-stimulus theta (p=0.39) but significantly greater iCoh in pre-stimulus alpha (p=0.04). No group effects emerged in post-stimulus theta or alpha iCoh (all p>0.10). For beta, there were significant differences between ADHD persisters and controls (p<0.001) and between remitters and controls (p=0.02), with both ADHD groups showing greater iCoh than the control group, but no differences between persisters and remitters (p=0.22).

In the analyses of local connectivity within left/right hemispheres, the three-way group-by-window-by-hemisphere and two-way group-by-hemisphere interactions were not significant for theta, alpha or beta iCoh (all p>0.60). Group differences thus did not vary in the two hemispheres. For beta, the two-way group-by-window interaction was further not significant (p=0.85), indicating that the group differences did not vary in the two time windows, in line with the whole-brain analysis. After removing these non-significant interactions, results of analyses on group differences in local connectivity within left/right hemispheres were consistent with those on whole-brain connectivity. Post-hoc group comparisons showed, for theta and alpha iCoh in the pre-stimulus window, that ADHD persisters had significantly greater iCoh than controls (p<0.02) but did not differ from remitters (p>0.29). Relative to controls, remitters showed no differences in pre-stimulus theta (p=0.55) but differences approaching statistical significance emerged in pre-stimulus alpha (p=0.05), indicating greater iCoh in remitters than controls. No group effects emerged in post-stimulus theta or alpha iCoh (all p>0.19). For beta iCoh, there were significant differences between ADHD persisters and controls (p<0.01), between remitters and controls (p=0.04), indicating greater iCoh in both ADHD groups compared to controls, but no differences between persisters and remitters (p=0.11).

The analyses of local connectivity between anterior, central and posterior cortical regions showed that the three-way group-by-window-by-region and two-way group-by-region interactions were not significant for theta, alpha or beta iCoh (all p>0.23), indicating that the group differences at all frequency bands were comparable across anterio-central, anterio-posterior and centro-posterior connectivity. For beta iCoh, the two-way group-by-window interaction was further not significant (p=0.50), indicating that the group differences did not vary in the two time windows, in line with the whole-brain analysis. After removing these non-significant interactions, post-hoc comparisons showed the same pattern of differences between groups of whole-brain iCoh measures. In pre-stimulus theta and alpha, ADHD persisters showed significantly greater iCoh than controls (all p<0.01) but no differences from remitters (all p>0.25). Compared to controls, remitters showed no differences in theta iCoh (p=0.44) but significantly greater alpha iCoh (p=0.01). No group effects emerged in post-stimulus theta or alpha (all p>0.48). For beta iCoh, significant differences emerged between ADHD persisters and controls (p<0.01) and between remitters and controls (p=0.02), with both ADHD groups showing greater iCoh than controls, but not between persisters and remitters (p=0.22).

The analyses of connectivity between the left/right hemispheres showed that the two-way group-by-window interaction was significant for theta and alpha (all p<0.02), but not for beta (0.92), in line with the analysis of whole-brain iCoh. Group differences on iCoh between the two hemispheres showed the same pattern of group differences yielded in the main analyses on whole-brain connectivity. Post-hoc group comparisons showed that, for theta and alpha iCoh in the pre-stimulus window, ADHD persisters showed significantly greater iCoh than controls (p<0.05) but did not differ from remitters (p>0.39). Relative to controls, remitters showed no differences in pre-stimulus theta (p=0.40) but differences approaching statistical significance emerged in pre-stimulus alpha (p=0.05), indicating greater iCoh in remitters than controls. No group effects emerged in post-stimulus theta or alpha (all p>0.30). For beta iCoh, there were significant differences between ADHD persisters and controls (p<0.01) and between remitters and controls (p=0.02), with both ADHD groups showing greater iCoh than controls, but no differences between persisters and remitters (p=0.16).

**SUPPLEMENTARY TABLES**

**Supplementary Table 1.** Descriptive statistics (mean and standard deviation [SD]) for study variables divided by group

|  | | **Controls** | | **ADHD-R** | | **ADHD-P** | |
| --- | --- | --- | --- | --- | --- | --- | --- |
| **THETA** | | **Mean** | **SD** | **Mean** | **SD** | **Mean** | **SD** |
| **Average clustering coefficient** | *Pre, Corr* | 0.058 | 0.011 | 0.061 | 0.009 | 0.066 | 0.014 |
|  | *Pre, Err* | 0.091 | 0.019 | 0.085 | 0.016 | 0.091 | 0.018 |
|  | *Post, Corr* | 0.097 | 0.021 | 0.092 | 0.015 | 0.095 | 0.022 |
|  | *Post, Err* | 0.127 | 0.026 | 0.119 | 0.023 | 0.118 | 0.025 |
| **Global efficiency** | *Pre, Corr* | 0.063 | 0.015 | 0.065 | 0.010 | 0.070 | 0.016 |
|  | *Pre, Err* | 0.096 | 0.023 | 0.089 | 0.017 | 0.095 | 0.020 |
|  | *Post, Corr* | 0.109 | 0.026 | 0.100 | 0.020 | 0.105 | 0.027 |
|  | *Post, Err* | 0.138 | 0.030 | 0.129 | 0.029 | 0.128 | 0.029 |
| **Path length** | *Pre, Corr* | 17.571 | 3.084 | 16.671 | 2.374 | 15.797 | 3.095 |
|  | *Pre, Err* | 11.504 | 2.281 | 12.270 | 2.246 | 11.571 | 2.304 |
|  | *Post, Corr* | 10.561 | 2.157 | 11.143 | 2.152 | 10.942 | 2.331 |
|  | *Post, Err* | 8.132 | 1.630 | 8.659 | 1.762 | 8.810 | 1.898 |
| **Diameter** | *Pre, Corr* | 30.563 | 4.579 | 28.348 | 3.892 | 27.499 | 5.175 |
|  | *Pre, Err* | 19.506 | 3.649 | 20.356 | 3.265 | 19.781 | 3.882 |
|  | *Post, Corr* | 21.238 | 3.910 | 21.084 | 3.558 | 21.155 | 3.643 |
|  | *Post, Err* | 15.307 | 2.904 | 15.729 | 2.714 | 16.192 | 3.139 |
| **Mean imaginary coherence** | *Pre, Corr* | 0.060 | 0.012 | 0.063 | 0.009 | 0.068 | 0.015 |
|  | *Pre, Err* | 0.094 | 0.020 | 0.087 | 0.017 | 0.093 | 0.019 |
|  | *Post, Corr* | 0.102 | 0.023 | 0.095 | 0.017 | 0.099 | 0.024 |
|  | *Post, Err* | 0.132 | 0.027 | 0.124 | 0.025 | 0.122 | 0.026 |
| **ALPHA** | | **Mean** | **SD** | **Mean** | **SD** | **Mean** | **SD** |
| **Average clustering coefficient** | *Pre, Corr* | 0.069 | 0.019 | 0.077 | 0.018 | 0.078 | 0.024 |
|  | *Pre, Err* | 0.099 | 0.026 | 0.092 | 0.017 | 0.100 | 0.025 |
|  | *Post, Corr* | 0.074 | 0.014 | 0.075 | 0.010 | 0.078 | 0.013 |
|  | *Post, Err* | 0.104 | 0.017 | 0.095 | 0.014 | 0.100 | 0.017 |
| **Global efficiency** | *Pre, Corr* | 0.082 | 0.030 | 0.094 | 0.033 | 0.092 | 0.035 |
|  | *Pre, Err* | 0.107 | 0.030 | 0.102 | 0.020 | 0.113 | 0.034 |
|  | *Post, Corr* | 0.081 | 0.018 | 0.082 | 0.015 | 0.084 | 0.016 |
|  | *Post, Err* | 0.111 | 0.019 | 0.100 | 0.015 | 0.106 | 0.018 |
| **Path length** | *Pre, Corr* | 14.516 | 3.913 | 12.714 | 3.416 | 13.230 | 4.125 |
|  | *Pre, Err* | 10.552 | 2.569 | 10.909 | 1.969 | 10.219 | 2.555 |
|  | *Post, Corr* | 13.795 | 2.544 | 13.363 | 2.035 | 13.169 | 2.364 |
|  | *Post, Err* | 9.907 | 1.678 | 10.850 | 1.524 | 10.294 | 1.730 |
| **Diameter** | *Pre, Corr* | 27.304 | 5.210 | 24.828 | 5.459 | 24.927 | 5.713 |
|  | *Pre, Err* | 18.570 | 3.770 | 19.499 | 3.374 | 18.534 | 3.988 |
|  | *Post, Corr* | 25.133 | 3.944 | 24.676 | 3.278 | 23.665 | 3.940 |
|  | *Post, Err* | 17.246 | 2.797 | 18.710 | 2.806 | 17.870 | 3.163 |
| **Mean imaginary coherence** | *Pre, Corr* | 0.073 | 0.022 | 0.081 | 0.021 | 0.082 | 0.027 |
|  | *Pre, Err* | 0.101 | 0.024 | 0.096 | 0.017 | 0.105 | 0.027 |
|  | *Post, Corr* | 0.077 | 0.015 | 0.078 | 0.011 | 0.081 | 0.014 |
|  | *Post, Err* | 0.107 | 0.018 | 0.097 | 0.014 | 0.103 | 0.017 |
| **BETA** | | **Mean** | **SD** | **Mean** | **SD** | **Mean** | **SD** |
| **Average clustering coefficient** | *Pre, Corr* | 0.056 | 0.009 | 0.060 | 0.009 | 0.065 | 0.013 |
|  | *Pre, Err* | 0.090 | 0.016 | 0.086 | 0.015 | 0.089 | 0.016 |
|  | *Post, Corr* | 0.057 | 0.008 | 0.061 | 0.009 | 0.064 | 0.014 |
|  | *Post, Err* | 0.091 | 0.016 | 0.087 | 0.016 | 0.089 | 0.017 |
| **Global efficiency** | *Pre, Corr* | 0.059 | 0.010 | 0.062 | 0.009 | 0.067 | 0.014 |
|  | *Pre, Err* | 0.092 | 0.016 | 0.088 | 0.016 | 0.091 | 0.017 |
|  | *Post, Corr* | 0.058 | 0.008 | 0.063 | 0.009 | 0.066 | 0.015 |
|  | *Post, Err* | 0.093 | 0.016 | 0.088 | 0.016 | 0.091 | 0.017 |
| **Path length** | *Pre, Corr* | 18.294 | 2.502 | 17.130 | 2.342 | 16.292 | 2.892 |
|  | *Pre, Err* | 11.648 | 2.054 | 12.183 | 2.151 | 11.783 | 2.028 |
|  | *Post, Corr* | 18.216 | 2.161 | 17.010 | 2.265 | 16.374 | 2.927 |
|  | *Post, Err* | 11.515 | 2.039 | 12.139 | 2.264 | 11.852 | 2.053 |
| **Diameter** | *Pre, Corr* | 30.381 | 3.795 | 28.345 | 3.480 | 26.796 | 4.702 |
|  | *Pre, Err* | 18.936 | 3.392 | 19.749 | 3.795 | 19.290 | 3.519 |
|  | *Post, Corr* | 29.972 | 3.522 | 28.124 | 4.187 | 26.857 | 4.818 |
|  | *Post, Err* | 18.726 | 3.295 | 19.627 | 3.623 | 19.506 | 3.549 |
| **Mean imaginary coherence** | *Pre, Corr* | 0.058 | 0.009 | 0.062 | 0.009 | 0.066 | 0.014 |
|  | *Pre, Err* | 0.092 | 0.016 | 0.087 | 0.015 | 0.091 | 0.017 |
|  | *Post, Corr* | 0.058 | 0.008 | 0.062 | 0.009 | 0.066 | 0.014 |
|  | *Post, Err* | 0.092 | 0.016 | 0.088 | 0.016 | 0.090 | 0.017 |

*Abbreviations: ADHD-P = ADHD persisters; ADHD-R = ADHD remitters; Corr = correctly-responded trials; Ctrl = Control group; Err = incorrectly-responded trials; Pre = pre-stimulus time window; Post = post-stimulus time window. Data in correctly-responded trials were available for 83 ADHD persisters, 22 remitters, 166 controls; and in incorrectly-responded trials for 75 ADHD persisters, 20 remitters, 145 controls.*

**Supplementary Table 2.** Full results of repeated measures mixed models, showing all main and interaction effects

| **THETA** | **Group** | **Window** | **Response** | **Group-by- Window** | **Group-by- Response** | **Group-by- Window-by-Response** |
| --- | --- | --- | --- | --- | --- | --- |
| **Average clustering coefficient** | 0.258 | <0.001*** | <0.001*** | 0.001** | 0.007** | 0.688 |
| **Global efficiency** | 0.176 | <0.001*** | <0.001*** | 0.001** | 0.045* | 0.673 |
| **Path length** | 0.509 | <0.001*** | <0.001*** | <0.001*** | 0.002** | 0.018* |
| **Diameter** | 0.307 | <0.001*** | <0.001*** | 0.001** | <0.001*** | 0.029* |
| **Mean imaginary coherence** | 0.232 | <0.001*** | <0.001*** | 0.001** | 0.014* | 0.676 |
| **ALPHA** | **Group** | **Window** | **Response** | **Group-by- Window** | **Group-by- Response** | **Group-by- Window-by-Response** |
| **Average clustering coefficient** | 0.392 | 0.114 | <0.001*** | 0.024* | <0.001*** | 0.853 |
| **Global efficiency** | 0.507 | 0.005** | <0.001*** | 0.002** | 0.005** | 0.721 |
| **Path length** | 0.305 | 0.507 | <0.001*** | 0.022* | <0.001*** | 0.713 |
| **Diameter** | 0.158 | <0.001*** | <0.001*** | 0.106 | <0.001*** | 0.642 |
| **Mean imaginary coherence** | 0.345 | 0.529 | <0.001*** | 0.004** | <0.001*** | 0.794 |
| **BETA** | **Group** | **Window** | **Response** | **Group-by- Window** | **Group-by- Response** | **Group-by- Window-by-Response** |
| **Average clustering coefficient** | 0.038* | 0.728 | <0.001*** | 0.856 | <0.001*** | 0.945 |
| **Global efficiency** | 0.047* | 0.904 | <0.001*** | 0.849 | <0.001*** | 0.887 |
| **Path length** | <0.001*** | 0.831 | <0.001*** | 0.8305 | <0.001*** | 0.989 |
| **Diameter** | <0.001*** | 0.695 | <0.001*** | 0.630 | <0.001*** | 0.997 |
| **Mean imaginary coherence** | 0.047* | 0.866 | <0.001*** | 0.864 | <0.001*** | 0.939 |

*Notes: Random intercept linear models tested for main effects of group (ADHD remitters vs ADHD persisters vs controls), time window (pre-stimulus vs post-stimulus) ad response (correctly- vs incorrectly-responded trials), two-way interactions (group-by-window, group-by-response, time window-by-response), and three-way interactions (group-by-window-by-response) on connectivity measures. Age was included as a covariate of no interest in all analyses. Data in correctly-responded trials were available for 83 ADHD persisters, 22 remitters, 166 controls; and in incorrectly-responded trials for 75 ADHD persisters, 20 remitters, 145 controls.*

**p<0.05; **p<0.01; ***p<0.001.*

**Supplementary Table 3.** Group comparisons on graph-theory and imaginary coherence measures in male participants only

|  | |  | **Group comparison** | | | | | | |
| --- | --- | --- | --- | --- | --- | --- | --- | --- | --- |
| **THETA** |  | **Overall Group** | **ADHD-P vs Ctrl** | | **ADHD-R vs Ctrl** | | **ADHD-R vs ADHD-P** | |  |
|  |  | **p** | **p** | **d** | **p** | **d** | **p** | **d** |  |
| **Average clustering coefficient** | *Pre, Corr* | 0.052 | 0.019* | *0.55* | 0.972 | 0.20 | 0.148 | 0.36 |  |
|  | *Pre, Err* | 0.642 | - | - | - | - | - | - |  |
|  | *Post, Corr* | 0.569 | - | - | - | - | - | - |  |
|  | *Post, Err* | 0.014* | 0.008** | 0.28 | 0.058 | 0.26 | 0.878 | 0.03 |  |
| **Global efficiency** | *Pre, Corr* | 0.129 | 0.061 | 0.43 | 0.803 | 0.08 | 0.156 | 0.38 |  |
|  | *Pre, Err* | 0.637 | - | - | - | - | - | - |  |
|  | *Post, Corr* | 0. 398 | - | - | - | - | - | - |  |
|  | *Post, Err* | 0.019* | 0.012* | 0.28 | 0.062 | 0.26 | 0.835 | 0.02 |  |
| **Path length** | *Pre, Corr* | <0.001*** | <0.001*** | 0.49 | 0.213 | 0.22 | 0.134 | 0.31 |  |
|  | *Pre, Err* | 0.540 | - | - | - | - | - | - |  |
|  | *Post, Corr* | 0.499 | - | - | - | - | - | - |  |
|  | *Post, Err* | 0.376 | - | - | - | - | - | - |  |
| **Diameter** | *Pre, Corr* | <0.001*** | <0.001*** | *0.54* | 0.051 | 0.38 | 0.330 | 0.19 |  |
|  | *Pre, Err* | 0.806 | - | - | - | - | - | - |  |
|  | *Post, Corr* | 0.975 | - | - | - | - | - | - |  |
|  | *Post, Err* | 0.578 | - | - | - | - | - | - |  |
| **Mean imaginary coherence** | *Pre, Corr* | 0.073 | 0.028* | *0.52* | 0.957 | 0.16 | 0.151 | 0.37 |  |
|  | *Pre, Err* | 0.654 | - | - | - | - | - | - |  |
|  | *Post, Corr* | 0.511 | - | - | - | - | - | - |  |
|  | *Post, Err* | 0.015* | 0.009** | 0.28 | 0.062 | 0.26 | 0.881 | 0.03 |  |
| **ALPHA** |  | **Overall Group** | **ADHD-P vs Ctrl** | | **ADHD-R vs Ctrl** | | **ADHD-R vs ADHD-P** | |  |
|  |  | **p** | **p** | **d** | **p** | **d** | **p** | **d** |  |
| **Average clustering coefficient** | *Pre, Corr* | 0.006** | 0.001** | 0.41 | 0.219 | 0.30 | 0.403 | 0.13 |  |
|  | *Pre, Err* | 0.217 | - | - | - | - | - | - |  |
|  | *Post, Corr* | 0.365 | - | - | - | - | - | - |  |
|  | *Post, Err* | 0.130 | - | - | - | - | - | - |  |
| **Global efficiency** | *Pre, Corr* | 0.013* | 0.004** | 0.31 | 0.146 | 0.28 | 0.678 | 0.04 |  |
|  | *Pre, Err* | 0.143 | - | - | - | - | - | - |  |
|  | *Post, Corr* | 0.871 | - | - | - | - | - | - |  |
|  | *Post, Err* | 0.283 | - | - | - | - | - | - |  |
| **Path length** | *Pre, Corr* | 0.004** | 0.003** | 0.28 | 0.027* | 0.36 | 0.824 | 0.06 |  |
|  | *Pre, Err* | 0.402 | - | - | - | - | - | - |  |
|  | *Post, Corr* | 0.334 | - | - | - | - | - | - |  |
|  | *Post, Err* | 0.426 | - | - | - | - | - | - |  |
| **Diameter** | *Corr* | 0.003** | 0.001** | 0.36 | 0.177 | 0.21 | 0.422 | 0.19 |  |
|  | *Err* | 0.524 | - | - | - | - | - | - |  |
| **Mean imaginary coherence** | *Pre, Corr* | 0.004** | 0.001** | 0.38 | 0.174 | 0.28 | 0.425 | 0.11 |  |
|  | *Pre, Err* | 0.126 | - | - | - | - | - | - |  |
|  | *Post, Corr* | 0.548 | - | - | - | - | - | - |  |
|  | *Post, Err* | 0.152 | - | - | - | - | - | - |  |
| **BETA** |  | **Overall Group** | **ADHD-P vs Ctrl** | | **ADHD-R vs Ctrl** | | **ADHD-R vs ADHD-P** | |  |
|  |  | **p** | **p** | **d** | **p** | **d** | **p** | **d** |  |
| **Average clustering coefficient** | *Corr* | <0.001*** | <0.001*** | *0.69* | 0.214 | 0.40 | 0.092 | 0.32 |  |
|  | *Err* | 0.433 | - | - | - | - | - | - |  |
| **Global efficiency** | *Corr* | <0.001*** | <0.001*** | *0.63* | 0.291 | 0.33 | 0.092 | 0.31 |  |
|  | *Err* | 0.431 | - | - | - | - | - | - |  |
| **Path length** | *Corr* | <0.001*** | <0.001*** | *0.66* | 0.027* | 0.41 | 0.091 | 0.27 |  |
|  | *Err* | 0.624 | - | - | - | - | - | - |  |
| **Diameter** | *Corr* | <0.001*** | <0.001*** | *0.76* | 0.024* | 0.42 | 0.042* | 0.32 |  |
|  | *Err* | 0.588 | - | - | - | - | - | - |  |
| **Mean imaginary coherence** | *Corr* | <0.001*** | <0.001*** | *0.67* | 0.214 | 0.38 | 0.092 | 0.31 |  |
|  | *Err* | 0.434 | - | - | - | - | - | - |  |

*Abbreviations: ADHD-P = ADHD persisters; ADHD-R = ADHD remitters; Corr = correctly-responded trials; Ctrl = Control group; d = Cohen’s d effect size; Err = incorrectly-responded trials; p = random intercept linear model significant testing; Pre = pre-stimulus time window; Post = post-stimulus time window.*

*Notes: Random intercept linear models tested for main effects of group (ADHD remitters vs ADHD persisters vs controls), time window (pre-stimulus vs post-stimulus) ad response (correctly- vs incorrectly-responded trials), two-way interactions (group-by-window, group-by-response, time window-by-response), and three-way interactions (group-by-window-by-response) on connectivity measures. Since neither diameter in the alpha band, nor any measures in the beta band showed a significant group-by-window interaction, post-hoc effects of group were tested with correctly- and incorrectly-responded trials collapsed across pre-stimulus and post-stimulus time windows. Post-hoc comparisons between groups were run only on measures showing a significant overall group effect. Age was included as a covariate of no interest in all analyses. Data in correctly-responded trials were available for 68 ADHD persisters, 22 remitters, 125 controls; and in incorrectly-responded trials for 63 ADHD persisters, 20 remitters, 110 controls. d≥0.20 = small effect size, d≥0.50 = medium effect (in italics), d≥0.80 = large effect.*

**p<0.05; **p<0.01; ***p<0.001.*

**Supplementary Table 4.** Within- and between-group effects on measures of change between pre-stimulus and post-stimulus windows in graph-theory and imaginary coherence measures in male participants only

|  |  | **Within-Group Change** | | | **Between-Group Change** | | | | | |
| --- | --- | --- | --- | --- | --- | --- | --- | --- | --- | --- |
| **THETA** |  | **Ctrl** | **ADHD-P** | **ADHD-R** | **ADHD-P vs Ctrl** | | **ADHD-R vs Ctrl** | | **ADHD-R vs ADHD-P** | |
|  |  | **p** | **p** | **p** | **p** | **d** | **p** | **d** | **p** | **d** |
| **Average clustering coefficient** | *Corr* | <0.001*** | <0.001*** | <0.001*** | 0.009* | 0.37 | 0.025* | 0.36 | 0.962 | 0.04 |
|  | *Err* | <0.001*** | <0.001*** | <0.001*** | 0.078 | 0.26 | 0.741 | 0.03 | 0.471 | 0.23 |
| **Global efficiency** | *Corr* | <0.001*** | <0.001*** | <0.001*** | 0.018* | 0.33 | 0.034* | 0.34 | 0.929 | 0.03 |
|  | *Err* | <0.001*** | <0.001*** | <0.001*** | 0.125 | 0.22 | 0.801 | 0.01 | 0.801 | 0.22 |
| **Path length** | *Corr* | <0.001*** | <0.001*** | <0.001*** | <0.001*** | *0.52* | 0.025* | 0.36 | 0.678 | 0.17 |
|  | *Err* | <0.001*** | <0.001*** | <0.001*** | 0.173 | 0.23 | 0.719 | 0.09 | 0.263 | 0.31 |
| **Diameter** | *Corr* | <0.001*** | <0.001*** | <0.001*** | <0.001*** | *0.51* | 0.061 | 0.33 | 0.491 | 0.20 |
|  | *Err* | <0.001*** | <0.001*** | <0.001*** | 0.200 | 0.20 | 0.755 | 0.12 | 0.256 | 0.30 |
| **Mean imaginary coherence** | *Corr* | <0.001*** | <0.001*** | <0.001*** | 0.012* | 0.35 | 0.027* | 0.36 | 0.941 | 0.03 |
|  | *Err* | <0.001*** | <0.001*** | <0.001*** | 0.089 | 0.25 | 0.765 | 0.02 | 0.480 | 0.22 |
| **ALPHA** |  | **Ctrl** | **ADHD-P** | **ADHD-R** | **ADHD-P vs Ctrl** | | **ADHD-R vs Ctrl** | | **ADHD-R vs ADHD-P** | |
|  |  | **p** | **p** | **p** | **p** | **d** | **p** | **d** | **p** | **d** |
| **Average clustering coefficient** | *Corr* | 0.031* | 0.849 | 0.770 | 0.108 | 0.25 | 0.136 | 0.32 | 0.807 | 0.04 |
|  | *Err* | 0.005** | 0.990 | 0.604 | 0.283 | 0.26 | 0.117 | 0.17 | 0.537 | 0.12 |
| **Global efficiency** | *Corr* | 0.362 | 0.003** | 0.050 | 0.108 | 0.27 | 0.203 | 0.32 | 0.848 | 0.04 |
|  | *Err* | 0.218 | 0.039* | 0.691 | 0.027* | 0.38 | 0.133 | 0.25 | 0.397 | 0.16 |
| **Path length** | *Corr* | 0.083 | 0.930 | 0.323 | 0.275 | 0.15 | 0.091 | 0.34 | 0.372 | 0.19 |
|  | *Err* | 0.024* | 0.753 | 0.931 | 0.025* | 0.38 | 0.087 | 0.32 | 0.700 | 0.09 |
| **Mean imaginary coherence** | *Corr* | 0.142 | 0.327 | 0.477 | 0.113 | 0.25 | 0.163 | 0.30 | 0.858 | 0.03 |
|  | *Err* | 0.001** | 0.581 | 0.793 | 0.021* | 0.40 | 0.064 | 0.32 | 0.517 | 0.12 |

*Abbreviations: ADHD-P = ADHD persisters; ADHD-R = ADHD remitters; Corr = correctly-responded trials; Ctrl = Control group; d = Cohen’s d effect size; Err = incorrectly-responded trials; p = random intercept linear model significance testing.*

*Notes: Random intercept linear models tested for main effects of group (ADHD remitters vs ADHD persisters vs controls), time window (pre-stimulus vs post-stimulus) ad response (correctly- vs incorrectly-responded trials), two-way interactions (group-by-window, group-by-response, time window-by-response), and three-way interactions (group-by-window-by-response) on connectivity measures. Post-hoc tests on within- and between-group effects of change were run only on measures showing a significant group-by-window interaction. Since in diameter in the alpha band and in all measures in the beta band this interaction was not significant, post-hoc within- and between-groups effects of change were not tested. Age was included as a covariate of no interest in all analyses. Data in correctly-responded trials were available for 68 ADHD persisters, 22 remitters, 125 controls; and in incorrectly-responded trials for 63 ADHD persisters, 20 remitters, 110 controls.* *d≥0.20 = small effect size, d≥0.50 = medium effect (in italics), d≥0.80 = large effect.*

**p<0.05; **p<0.01; ***p<0.001.*

**Supplementary Table 5.** Group comparisons on graph-theory and imaginary coherence measures covarying for IQ

|  | |  | **Group comparison** | | | | | | |
| --- | --- | --- | --- | --- | --- | --- | --- | --- | --- |
| **THETA** |  | **Overall Group** | **ADHD-P vs Ctrl** | | **ADHD-R vs Ctrl** | | **ADHD-R vs ADHD-P** | | |
|  |  | **p** | **p** | **d** | **p** | **d** | **p** | **d** |  |
| **Average clustering coefficient** | *Pre, Corr* | 0.002** | <0.001*** | 0.46 | 0.690 | 0.20 | 0.086 | 0.25 |  |
|  | *Pre, Err* | 0.501 | - | - | - | - | - | - |  |
|  | *Post, Corr* | 0.649 | - | - | - | - | - | - |  |
|  | *Post, Err* | 0.004** | 0.003** | 0.11 | 0.030** | 0.22 | 0.759 | 0.12 |  |
| **Global efficiency** | *Pre, Corr* | 0.011* | 0.003** | 0.36 | 0.923 | 0.09 | 0.095 | 0.28 |  |
|  | *Pre, Err* | 0.502 | - | - | - | - | - | - |  |
|  | *Post, Corr* | 0.442 | - | - | - | - | - | - |  |
|  | *Post, Err* | 0.005** | 0.004** | 0.12 | 0.032* | 0.23 | 0.743 | 0.11 |  |
| **Path length** | *Pre, Corr* | <0.001*** | <0.001*** | 0.42 | 0.068 | 0.24 | 0.097 | 0.20 |  |
|  | *Pre, Err* | 0.441 | - | - | - | - | - | - |  |
|  | *Post, Corr* | 0.591 | - | - | - | - | - | - |  |
|  | *Post, Err* | 0.320 | - | - | - | - | - | - |  |
| **Diameter** | *Pre, Corr* | <0.001*** | <0.001*** | *0.50* | 0.009** | 0.43 | 0.298 | 0.09 |  |
|  | *Pre, Err* | 0.735 | - | - | - | - | - | - |  |
|  | *Post, Corr* | 0.848 | - | - | - | - | - | - |  |
|  | *Post, Err* | 0.503 | - | - | - | - | - | - |  |
| **Mean imaginary coherence** | *Pre, Corr* | 0.004** | 0.001** | 0.43 | 0.762 | 0.17 | 0.087 | 0.26 |  |
|  | *Pre, Err* | 0.505 | - | - | - | - | - | - |  |
|  | *Post, Corr* | 0.594 | - | - | - | - | - | - |  |
|  | *Post, Err* | 0.005** | 0.004** | 0.11 | 0.032* | 0.22 | 0.764 | 0.11 |  |
| **ALPHA** |  | **Overall Group** | **ADHD-P vs Ctrl** | | **ADHD-R vs Ctrl** | | **ADHD-R vs ADHD-P** | | |
|  |  | **p** | **p** | **d** | **p** | **d** | **p** | **d** |  |
| **Average clustering coefficient** | *Pre, Corr* | 0.001** | <0.001*** | 0.37 | 0.078 | 0.38 | 0.567 | 0.02 |  |
|  | *Pre, Err* | 0.353 | - | - | - | - | - | - |  |
|  | *Post, Corr* | 0.227 | - | - | - | - | - | - |  |
|  | *Post, Err* | 0.090 | - | - | - | - | - | - |  |
| **Global efficiency** | *Pre, Corr* | 0.003** | 0.001** | 0.28 | 0.044* | 0.37 | 0.954 | 0.07 |  |
|  | *Pre, Err* | 0.247 | - | - | - | - | - | - |  |
|  | *Post, Corr* | 0.685 | - | - | - | - | - | - |  |
|  | *Post, Err* | 0.228 | - | - | - | - | - | - |  |
| **Path length** | *Pre, Corr* | <0.001*** | <0.001*** | 0.29 | 0.004** | 0.45 | 0.626 | 0.15 |  |
|  | *Pre, Err* | 0.506 | - | - | - | - | - | - |  |
|  | *Post, Corr* | 0.117 | - | - | - | - | - | - |  |
|  | *Post, Err* | 0.355 | - | - | - | - | - | - |  |
| **Diameter** | *Corr* | <0.001*** | <0.001*** | 0.36 | 0.036* | 0.28 | 0.497 | 0.10 |  |
|  | *Err* | 0.581 | - | - | - | - | - | - |  |
| **Mean imaginary coherence** | *Pre, Corr* | 0.001** | <0.001*** | 0.34 | 0.058 | 0.36 | 0.608 | <0.01 |  |
|  | *Pre, Err* | 0.250 | - | - | - | - | - | - |  |
|  | *Post, Corr* | 0.350 | - | - | - | - | - | - |  |
|  | *Post, Err* | 0.114 | - | - | - | - | - | - |  |
| **BETA** |  | **Overall Group** | **ADHD-P vs Ctrl** | | **ADHD-R vs Ctrl** | | **ADHD-R vs ADHD-P** | | |
|  |  | **p** | **p** | **d** | **p** | **d** | **p** | **d** |  |
| **Average clustering coefficient** | *Corr* | <0.001*** | <0.001*** | *0.57* | 0.073 | 0.39 | 0.075 | 0.21 |  |
|  | *Err* | 0.254 | - | - | - | - | - | - |  |
| **Global efficiency** | *Corr* | <0.001*** | <0.001*** | *0.53* | 0.108 | 0.33 | 0.075 | 0.22 |  |
|  | *Err* | 0.267 | - | - | - | - | - | - |  |
| **Path length** | *Corr* | <0.001*** | <0.001*** | *0.56* | 0.003** | 0.42 | 0.082 | 0.16 |  |
|  | *Err* | 0.432 | - | - | - | - | - | - |  |
| **Diameter** | *Corr* | <0.001*** | <0.001*** | *0.61* | 0.003** | 0.43 | 0.042* | 0.20 |  |
|  | *Err* | 0.299 | - | - | - | - | - | - |  |
| **Mean imaginary coherence** | *Corr* | <0.001*** | <0.001*** | *0.56* | 0.080 | 0.38 | 0.074 | 0.21 |  |
|  | *Err* | 0.261 | - | - | - | - | - | - |  |

*Abbreviations: ADHD-P = ADHD persisters; ADHD-R = ADHD remitters; Corr = correctly-responded trials; Ctrl = Control group; d = Cohen’s d effect size; Err = incorrectly-responded trials; p = random intercept linear model significance testing; Pre = pre-stimulus time window; Post = post-stimulus time window.*

*Notes: Random intercept linear models tested for main effects of group (ADHD remitters vs ADHD persisters vs controls), time window (pre-stimulus vs post-stimulus) ad response (correctly- vs incorrectly-responded trials), two-way interactions (group-by-window, group-by-response, time window-by-response), and three-way interactions (group-by-window-by-response) on connectivity measures. Since neither diameter in the alpha band nor any measures in the beta band showed a significant group-by-window interaction, post-hoc effects of group were tested for with correctly- and incorrectly-responded trials collapsed across pre-stimulus and post-stimulus time windows. Post-hoc comparisons between groups were run only on measures showing a significant overall group effect. Age was included as a covariate of no interest in all analyses. Data in correctly-responded trials were available for 83 ADHD persisters, 22 remitters, 166 controls; and in incorrectly-responded trials for 75 ADHD persisters, 20 remitters, 145 controls. d≥0.20 = small effect size, d≥0.50 = medium effect (in italics), d≥0.80 = large effect.*

**p<0.05; **p<0.01; ***p<0.001.*

**Supplementary Table 6.** Within- and between-group effects on measures of change between pre-stimulus and post-stimulus windows in graph-theory and imaginary coherence measures covarying for IQ

|  |  | **Within-Group Change** | | | **Between-Group Change** | | | | | |
| --- | --- | --- | --- | --- | --- | --- | --- | --- | --- | --- |
| **THETA** |  | **Ctrl** | **ADHD-R** | **ADHD-P** | **ADHD-P vs Ctrl** | | **ADHD-R vs Ctrl** | | **ADHD-R vs ADHD-P** | |
|  |  | **p** | **p** | **p** | **p** | **d** | **p** | **d** | **p** | **d** |
| **Average clustering coefficient** | *Corr* | <0.001*** | <0.001*** | <0.001*** | 0.021* | 0.21 | 0.022* | 0.32 | 0.747 | 0.07 |
|  | *Err* | <0.001*** | <0.001*** | <0.001*** | 0.120 | 0.14 | 0.770 | <0.01 | 0.557 | 0.14 |
| **Global efficiency** | *Corr* | <0.001*** | <0.001*** | <0.001*** | 0.041* | 0.19 | 0.033* | 0.30 | 0.718 | 0.08 |
|  | *Err* | <0.001*** | <0.001*** | <0.001*** | 0.161 | 0.13 | 0.793 | <0.01 | 0.589 | 0.13 |
| **Path length** | *Corr* | <0.001*** | <0.001*** | <0.001*** | <0.001*** | 0.39 | 0.025* | 0.35 | 0.678 | 0.06 |
|  | *Err* | <0.001*** | <0.001*** | <0.001*** | 0.173 | 0.17 | 0.719 | 0.14 | 0.263 | 0.29 |
| **Diameter** | *Corr* | <0.001*** | <0.001*** | <0.001*** | <0.001*** | 0.43 | 0.021* | 0.36 | 0.567 | 0.09 |
|  | *Err* | <0.001*** | <0.001*** | <0.001*** | 0.273 | 0.12 | 0.630 | 0.17 | 0.238 | 0.28 |
| **Mean imaginary coherence** | *Corr* | <0.001*** | <0.001*** | <0.001*** | 0.027* | 0.20 | 0.024* | 0.31 | 0.730 | 0.08 |
|  | *Err* | <0.001*** | <0.001*** | <0.001*** | 0.130 | 0.14 | 0.784 | <0.01 | 0.566 | 0.14 |
| **ALPHA** |  | **Ctrl** | **ADHD-R** | **ADHD-P** | **ADHD-P vs Ctrl** | | **ADHD-R vs Ctrl** | | **ADHD-R vs ADHD-P** | |
|  |  | **p** | **p** | **p** | **p** | **d** | **p** | **d** | **p** | **d** |
| **Average clustering coefficient** | *Corr* | 0.002** | 0.767 | 0.910 | 0.086 | 0.23 | 0.092 | 0.36 | 0.710 | 0.11 |
|  | *Err* | 0.001** | 0.599 | 0.981 | 0.261 | 0.16 | 0.396 | 0.12 | 0.696 | 0.05 |
| **Global efficiency** | *Corr* | 0.728 | 0.045* | 0.004** | 0.110 | 0.23 | 0.143 | 0.38 | 0.716 | 0.12 |
|  | *Err* | 0.155 | 0.683 | 0.029* | 0.072 | 0.27 | 0.193 | 0.21 | 0.510 | 0.08 |
| **Path length** | *Corr* | 0.002** | 0.319 | 0.856 | 0.127 | 0.17 | 0.047* | 0.40 | 0.378 | 0.24 |
|  | *Err* | 0.011* | 0.931 | 0.831 | 0.075 | 0.27 | 0.118 | 0.29 | 0.849 | <0.01 |
| **Mean imaginary coherence** | *Corr* | 0.020* | 0.472 | 0.491 | 0.096 | 0.23 | 0.111 | 0.35 | 0.746 | 0.11 |
|  | *Err* | 0.001** | 0.791 | 0.546 | 0.061 | 0.27 | 0.129 | 0.25 | 0.632 | 0.05 |

*Abbreviations: ADHD-P = ADHD persisters; ADHD-R = ADHD remitters; Corr = trials with correct responses; Ctrl = Control group; d = Cohen’s d effect size; Err = trials with incorrect responses; p = random intercept linear model significance testing.*

*Notes: Random intercept linear models tested for main effects of group (ADHD remitters vs ADHD persisters vs controls), time window (pre-stimulus vs post-stimulus) ad response (correctly- vs incorrectly- responded trials), two-way interactions (group-by-window, group-by-response, time window-by-response), and three-way interactions (group-by-window-by-response) on connectivity measures. Post-hoc tests on within- and between-group effects of change were run only on measures showing a significant group-by-time window interaction. Since this interaction was not significant in diameter in the alpha band and in all measures in the beta band, post-hoc within- and between-groups effects of change were not tested. Age was also included as a covariate of no interest in all analyses. Data in correctly-responded trials were available for 83 ADHD persisters, 22 remitters, 166 controls; and in incorrectly-responded trials for 75 ADHD persisters, 20 remitters, 145 controls. d≥0.20 = small effect size, d≥0.50 = medium effect (in italics) and d≥0.80 = large effect size.*

**p<0.05; **p<0.01; ***p<0.001.*

**Supplementary Table 7.** Dimensional associations between graph-theory and imaginary coherence measures and interview-based DIVA ADHD symptom counts and clinical impairment within the childhood ADHD group only, controlling for age, gender and IQ

| **THETA** |  | **ADHD symptoms** | | **Impairment** | |
| --- | --- | --- | --- | --- | --- |
|  |  | **β (95% CIs)** | **p** | **β (95% CIs)** | **p** |
| **Average clustering coefficient** | *Pre, Corr* | -0.021 (-0.227;0.186) | 0.844 | 0.159 (-0.075;0.394) | 0.183 |
|  | *Pre, Err* | 0.036 (-0.171;0.243) | 0.734 | 0.213 (-0.013;0.439) | 0.064 |
|  | *Post, Corr* | -0.040 (-0.241;0.161) | 0.696 | 0.127 (-0.108;0.363) | 0.290 |
|  | *Post, Err* | -0.080 (-0.246;0.086) | 0.343 | -0.023 (-0.226;0.180) | 0.823 |
| **Global efficiency** | *Pre, Corr* | -0.045 (-0.232;0.142) | 0.637 | 0.109 (-0.105;0.323) | 0.318 |
|  | *Pre, Err* | 0.018 (-0.177;0.213) | 0.856 | 0.197 (-0.016;0.411) | 0.069 |
|  | *Post, Corr* | -0.061 (-0.258;0.136) | 0.547 | 0.066 (-0.168;0.301) | 0.580 |
|  | *Post, Err* | -0.103 (-0.265;0.058) | 0.210 | -0.141 (-0.338;0.055) | 0.159 |
| **Path length** | *Pre, Corr* | 0.045 (-0.140;0.231) | 0.632 | -0.142 (-0.351;0.067) | 0.182 |
|  | *Pre, Err* | -0.048 (-0.263;0.167) | 0.664 | -0.211 (-0.447;0.025) | 0.079 |
|  | *Post, Corr* | 0.074 (-0.139;0.287) | 0.496 | -0.056 (-0.307;0.195) | 0.662 |
|  | *Post, Err* | 0.083 (-0.121;0.286) | 0.425 | -0.003 (-0.238;0.233) | 0.983 |
| **Diameter** | *Pre, Corr* | 0.060 (-0.131;0.250) | 0.540 | -0.115 (-0.336;0.106) | 0.308 |
|  | *Pre, Err* | -0.060 (-0.278;0.158) | 0.589 | -0.198 (-0.437;0.041) | 0.104 |
|  | *Post, Corr* | 0.031 (-0.167;0.230) | 0.756 | -0.128(-0.351;0.096) | 0.262 |
|  | *Post, Err* | 0.072 (-0.134;0.278) | 0.495 | -0.026 (-0.256;0.204) | 0.825 |
| **Mean imaginary coherence** | *Pre, Corr* | -0.026 (-0.228;0.175) | 0.799 | 0.148 (-0.082;0.377) | 0.207 |
|  | *Pre, Err* | 0.031 (-0.173;0.234) | 0.768 | 0.209 (-0.014;0.431) | 0.066 |
|  | *Post, Corr* | -0.047 (-0.248;0153) | 0.642 | 0.108 (-0.013;0.344) | 0.370 |
|  | *Post, Err* | -0.090 (-0.253;0.073) | 0.280 | 0.056 (-0.266;0.137) | 0.531 |
| **ALPHA** |  | **ADHD symptoms** | | **Impairment** | |
|  |  | **β (95% CIs)** | **p** | **β (95% CIs)** | **p** |
| **Average clustering coefficient** | *Pre, Corr* | 0.011 (-0.198;0.220) | 0.918 | 0.056 (-0.188;0.300) | 0.653 |
|  | *Pre, Err* | 0.042 (-0.148;0.232) | 0.666 | 0.158 (-0.058;0.374) | 0.151 |
|  | *Post, Corr* | 0.024 (-0.165;0.213) | 0.804 | 0.222 (0.013;0.431) | 0.037* |
|  | *Post, Err* | 0.128 (-0.063;0.319) | 0.190 | 0.254 (0.040;0.467) | 0.020* |
| **Global efficiency** | *Pre, Corr* | -0.055 (-0.261;0.150) | 0.596 | -0.069 (-0.318;0.180) | 0.587 |
|  | *Pre, Err* | 0.025 (-0.177;0.227) | 0.807 | 0.135 (-0.096;0.366) | 0.251 |
|  | *Post, Corr* | 0.017 (-0.164;0.199) | 0.852 | 0.166 (-0.038;0.369) | 0.111 |
|  | *Post, Err* | 0.115 (-0.070;0.298) | 0.221 | 0.245 (0.038;0.451) | 0.020* |
| **Path length** | *Pre, Corr* | -0.004 (-0.190;0.183) | 0.969 | 0.055 (-0.168;0.277) | 0.629 |
|  | *Pre, Err* | -0.016 (-0.217;0.185) | 0.876 | -0.133 (-0.358;0.092) | 0.246 |
|  | *Post, Corr* | -0.022 (-0.212;0.168) | 0.819 | -0.182 (-0.392;0.029) | 0.090 |
|  | *Post, Err* | -0.127 (-0.326;0.071) | 0.208 | -0.239 (-0.462;-0.016) | 0.036* |
| **Diameter** | *Pre, Corr* | -0.033 (-0.237;0.171) | 0.749 | -0.049 (-0.285;0.188) | 0.688 |
|  | *Pre, Err* | -0.077 (-0.283;0.128) | 0.461 | -0.196 (-0.425;0.033) | 0.093 |
|  | *Post, Corr* | -0.032 (-0.230;0.166) | 0.751 | -0.225 (-0.444;-0.007) | 0.043* |
|  | *Post, Err* | -0.136 (-0.345;0.071) | 0.199 | -0.233 (-0.469;0.003) | 0.053 |
| **Mean imaginary coherence** | *Pre, Corr* | -0.004 (-0.209;0.201) | 0.971 | 0.015 (-0.226;0.255) | 0.905 |
|  | *Pre, Err* | 0.040 (-0.173;0.247) | 0.726 | 0.165 (-0.073;0.404) | 0.175 |
|  | *Post, Corr* | 0.0231 (-0.164;0.210) | 0.810 | 0.208 (0.001;0.415) | 0.049* |
|  | *Post, Err* | 0.125 (-0.064;0.315) | 0.195 | 0.252 (0.040;0.463) | 0.020* |
| **BETA** |  | **ADHD symptoms** | | **Impairment** | |
|  |  | **β (95% CIs)** | **p** | **β (95% CIs)** | **p** |
| **Average clustering coefficient** | *Pre, Corr* | 0.105 (-0.125;0.336) | 0.370 | 0.283 (0.029;0.539) | 0.029* |
|  | *Pre, Err* | 0.067 (-0.144;0.278) | 0.534 | 0.250 (0.018;0.482) | 0.035* |
|  | *Post, Corr* | 0.067 (-0.171;0.306) | 0.581 | 0.232 (-0.041;0.505) | 0.096 |
|  | *Post, Err* | 0.059 (-0.156;0.273) | 0.592 | 0.206 (-0.029;0.441) | 0.086 |
| **Global efficiency** | *Pre, Corr* | 0.090 (-0.135;0.314) | 0.433 | 0.282 (0.033;0.532) | 0.026* |
|  | *Pre, Err* | 0.061 (-0.151;0.273) | 0.572 | 0.256 (0.023;0.489) | 0.031* |
|  | *Post, Corr* | 0.048 (-0.192;0.287) | 0.696 | 0.224 (-0.050;0.449) | 0.110 |
|  | *Post, Err* | 0.054 (-0.160;0.269) | 0.618 | 0.205 (-0.030;0.441) | 0.087 |
| **Path length** | *Pre, Corr* | -0.083 (-0.284;0.118) | 0.419 | -0.220 (-0.446;0.006) | 0.057 |
|  | *Pre, Err* | -0.076 (-0.284;0.132) | 0.474 | -0.235 (-0.464;-0.006) | 0.044* |
|  | *Post, Corr* | -0.049 (-0.262;0.163) | 0.649 | -0.170 (-0.414;0.073) | 0.171 |
|  | *Post, Err* | -0.082 (-0.295;0.130) | 0.446 | -0.186 (-0.419;0.046) | 0.116 |
| **Diameter** | *Pre, Corr* | -0.111 (-0.316;0.093) | 0.286 | -0.238 (-0.468;-0.008) | 0.042* |
|  | *Pre, Err* | -0.106 (-0.323;0.109) | 0.335 | -0.200 (-0.439;0.038) | 0.100 |
|  | *Post, Corr* | -0.071 (-0.283;0.141) | 0.510 | -0.156 (-0.400;0.088) | 0.210 |
|  | *Post, Err* | -0.088 (-0.308;0.131) | 0.430 | -0.146 (-0.388;0.097) | 0.238 |
| **Mean imaginary coherence** | *Pre, Corr* | 0.101 (-0.128;0.330) | 0.389 | 0.284 (0.031;0.538) | 0.028* |
|  | *Pre, Err* | 0.065 (-0.146;0.277) | 0.544 | 0.253 (0.021;0.486) | 0.033* |
|  | *Post, Corr* | 0.061 (-0.179;0.301) | 0.620 | 0.229 (-0.046;0.504) | 0.102 |
|  | *Post, Err* | 0.057 (-0.158;0.271) | 0.604 | 0.206 (-0.030;0.441) | 0.087 |

*Abbreviations: β = standardized regression coefficient; CIs = confidence intervals; Corr = correctly-responded trials; Err = in correctly-responded trials; p = random intercept linear model significance testing; Pre = pre-stimulus time window; Post = post-stimulus time window.*

*Notes: Random intercept linear models tested the effect of ADHD symptoms and impairment on each connectivity measure, accounting for sibling relatedness. Data in correctly-responded trials were available for 105 childhood ADHD participants (83 ADHD persisters, 22 remitters); and in incorrectly-responded trials for 95 childhood ADHD participants (75 ADHD persisters, 20 remitters). β≥0.20 = small effect size, β≥0.50 = medium effect, β≥0.80 = large effect.*

**p<0.05.*

**Supplementary Table 8.** Association between pre-stimulus imaginary coherence measures in correctly-responded trials and task performance in the childhood ADHD and control groups, controlling for age and gender.

|  | | **Mean Reaction Time** | | **Reaction Time Variability** | | **Errors** | |
| --- | --- | --- | --- | --- | --- | --- | --- |
|  |  | **β (95% CIs)** | **p** | **β (95% CIs)** | **p** | **β (95% CIs)** | **p** |
| **Theta mean imaginary coherence** | **Childhood ADHD** | -0.030  (-0.211;0.151) | 0.746 | 0.221  (0.115;0.327) | <0.001*** | 0.070  (0.025;0.115) | 0.002** |
|  | **Control** | -0.121  (-0.287;0.045) | 0.153 | 0.073  (-0.018;0.166) | 0.116 | 0.057  (0.015;0.100) | 0.008** |
| **Alpha mean imaginary coherence** | **Childhood ADHD** | -0. 498  (-0.220;0.121) | 0.567 | 0.107  (-0.004;0.218) | 0.059 | 0.014  (-0.033;0.060) | 0.567 |
|  | **Control** | 0.120  (-0.044;0.283) | 0.151 | 0.078  (-0.012;0.168) | 0.090 | <0.001  (-0.042;0.042) | 0.999 |
| **Beta mean imaginary coherence** | **Childhood ADHD** | -0.063  (-0.202;0.077) | 0.380 | 0.230  (0.140;0.322) | <0.001*** | 0.092  (0.054;0.131) | <0.001*** |
|  | **Control** | -0.039  (-0.241;0.163) | 0.704 | 0.072  (-0.040;0.183) | 0.207 | 0.080  (0.029;0.131) | 0.002** |

*Abbreviations: β = standardized regression coefficient; CIs = confidence intervals; p = random intercept linear model significance testing.*

*Notes: Random intercept linear models tested the effect of ADHD symptoms and impairment on each connectivity measure, accounting for sibling relatedness. Data in correctly-responded trials were available for 105 childhood ADHD participants (83 ADHD persisters, 22 remitters) and 166 controls. β≥0.20 = small effect size, β≥0.50 = medium effect, β≥0.80 = large effect.*

***p<0.01; ***p<0.001.*

**
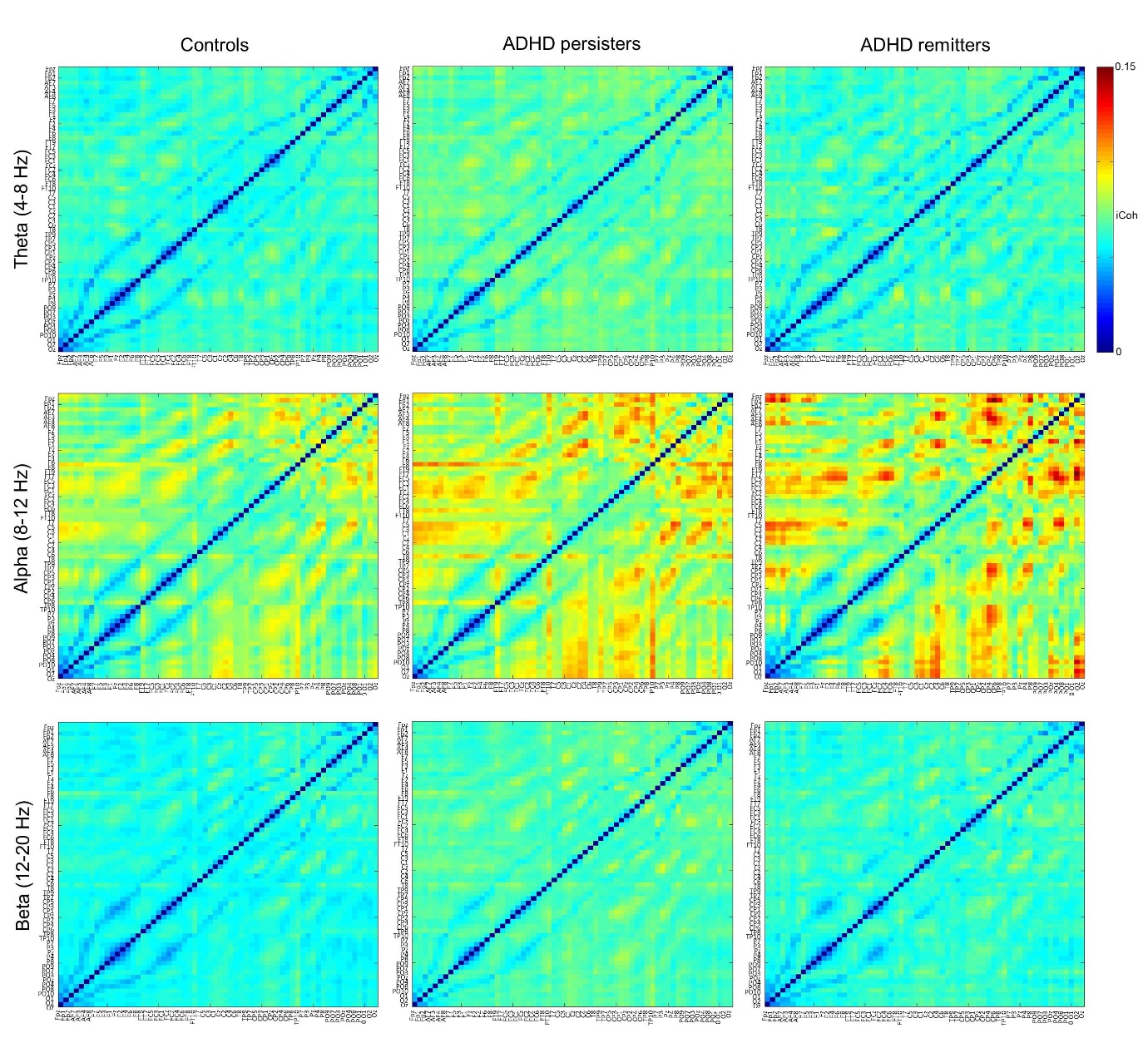
Supplementary Figure 1.** Connectivity matrices showing values of imaginary part of coherence (iCoh) in pre-stimulus theta, alpha and beta frequencies for correctly-responded trials by group (ADHD persisters, remitters and controls).

**
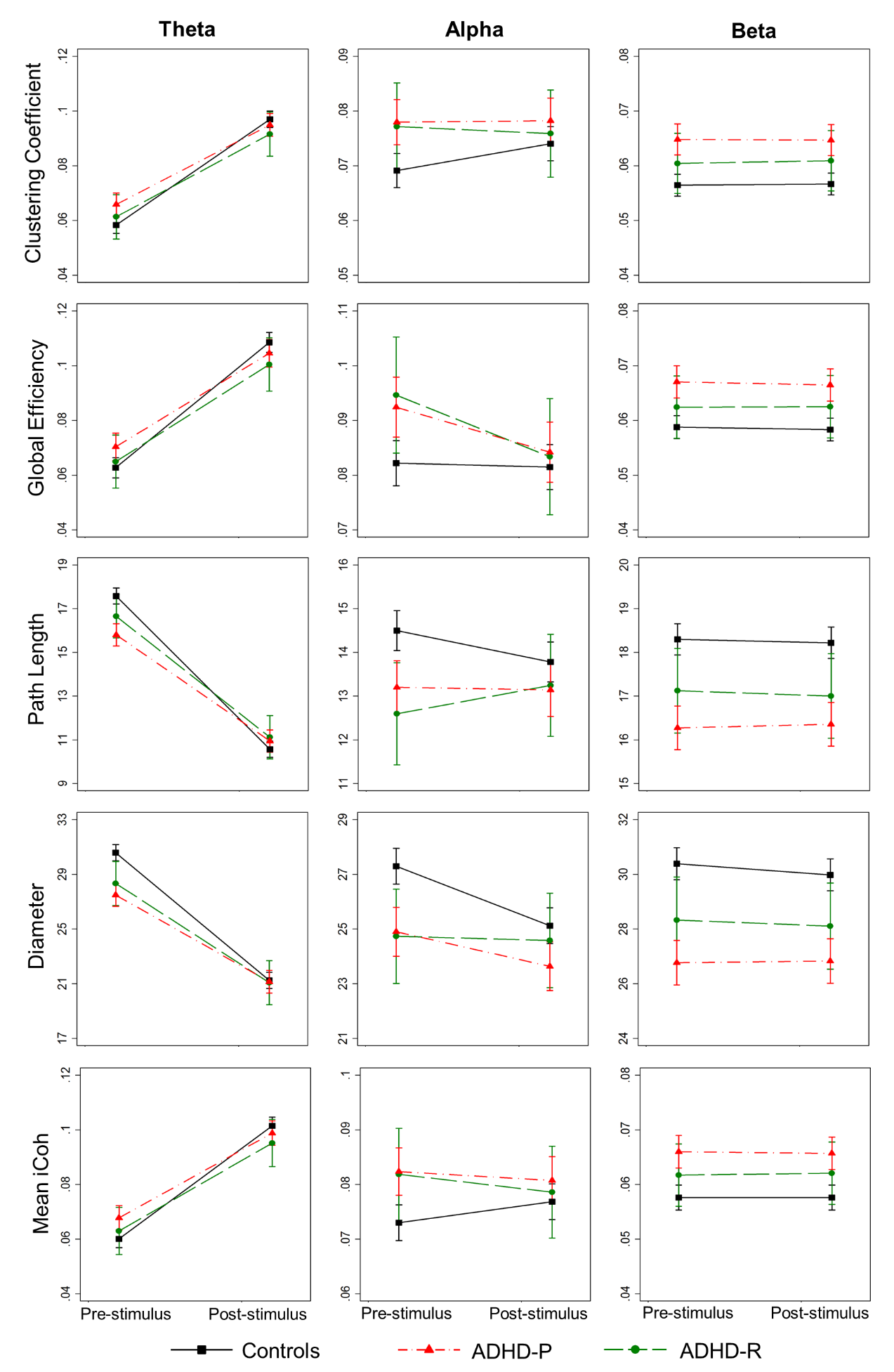
Supplementary Figure 2**. Results of the categorical analyses comparing ADHD persisters, remitters and controls on measures of graph theory and imaginary part of the coherence (iCoh) in the theta, alpha and beta band for correctly-responded trials.

**
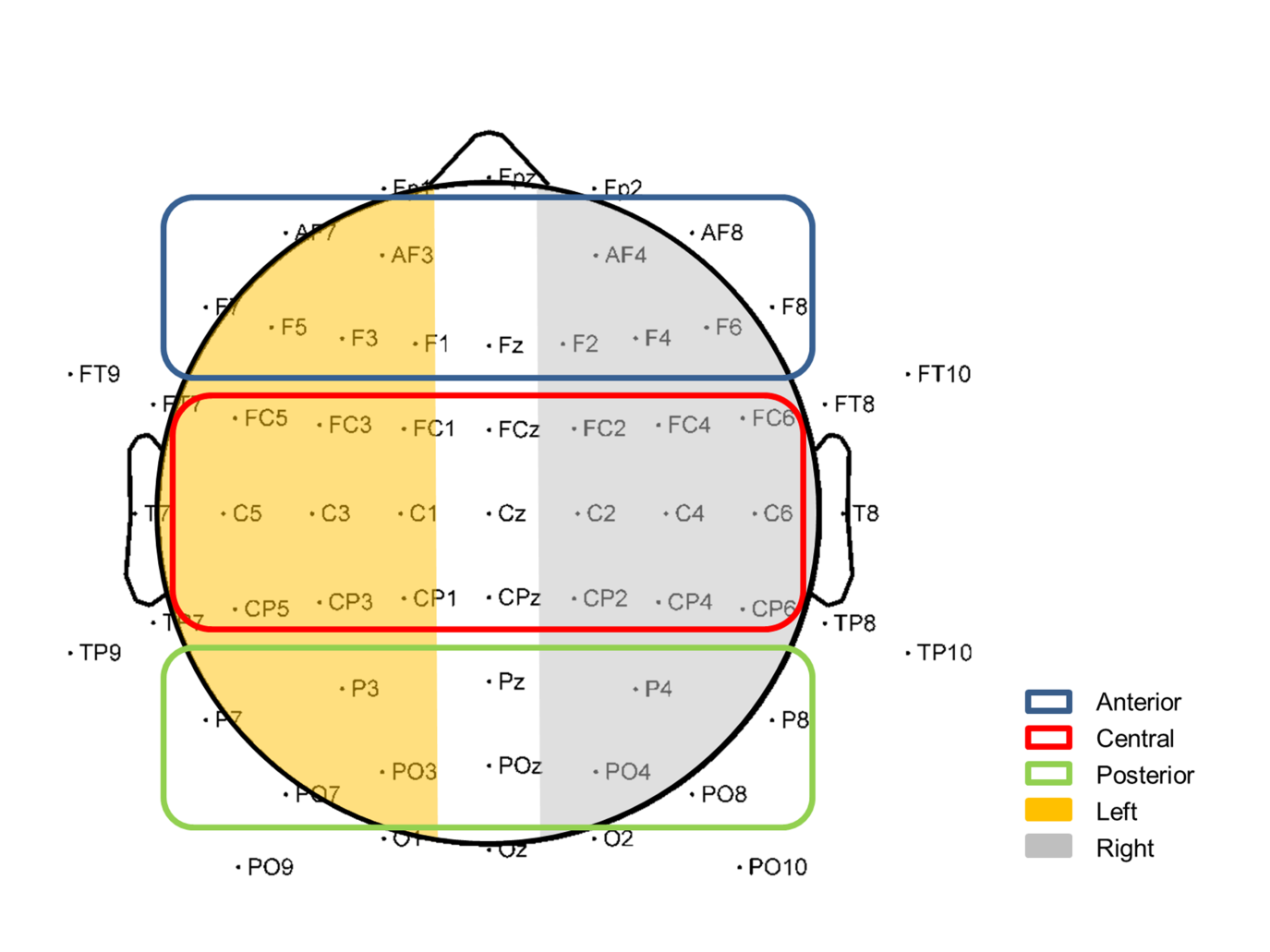
**

**Supplementary Figure 3.** Clusters of electrodes for local connectivity analysis.

**REFERENCES**

1. Cheung CH, Rijsdijk F, McLoughlin G, Brandeis D, Banaschewski T, Asherson P*, et al*. Cognitive and neurophysiological markers of ADHD persistence and remission. *Br J Psychiatry* 2016; **208**(6)**:** 548-555.

2. Cheung CH, Rijsdijk F, McLoughlin G, Faraone SV, Asherson P, Kuntsi J. Childhood predictors of adolescent and young adult outcome in ADHD. *J Psychiatr Res* 2015; **62:** 92-100.
